# Controlled Removal of Organic Dyes from Aqueous Systems Using Porous Cross-Linked Conjugated Polyanilines

**DOI:** 10.1021/acsapm.2c01718

**Published:** 2022-12-19

**Authors:** Julia C. Maxwell, Benjamin C. Baker, Charl F. J. Faul

**Affiliations:** School of Chemistry, University of Bristol, Cantock’s Close, Bristol BS8 1TS, UK

**Keywords:** porous materials, water purification, dye absorption, dye release, conjugated microporous polymers

## Abstract

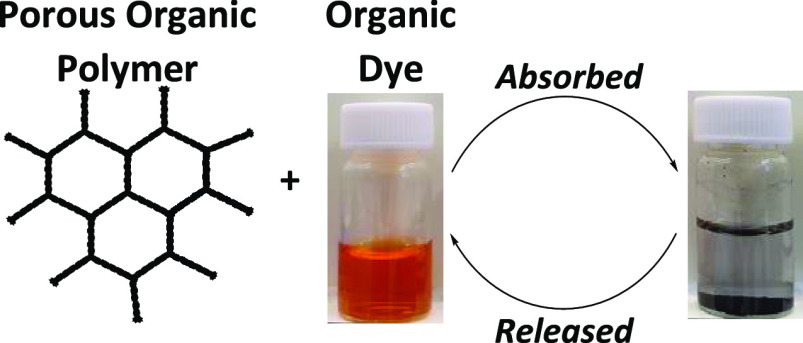

Porous organic materials, as a broad class of functional materials, offer a promising route for low-cost purification of contaminated wastewaters. We have synthesized a range of highly cross-linked conjugated porous polyanilines and optimized their porosity and water dispersibility by tuning reactant feed ratios, previously unreported in the synthesis of such networks. To demonstrate their ability to adsorb model dyes used in the textile industry, we exposed the networks to a range of cationic aromatic dyes, leading to absorption capacities of >100 mg/g, reported for the first time with respect to polyaniline networks. The versatility of the networks was further demonstrated by the preparation of gel composites, producing active gels for efficient and facile removal and recycling, ideal for real-world applications. Finally, chemical modifications of the networks were undertaken to target the removal of model anionic organic dye pollutants, showing the wide applicability of our approach.

## Introduction

Access to clean water is recognized as a key global goal for sustainable development under the United Nations Sustainable Development 2021 manifesto.^1^ Despite the fact that it is considered to be a basic human right, only one in three humans have access to safe drinking water globally,^2^ disproportionately affecting the lives of the vulnerable.^1,3^ More than 80% of wastewater resulting from human activities is discharged into rivers, lakes, or the sea without any treatment to remove pollutants.^4^

One class of these pollutants includes organic dyes, typically highly pigmented organic compounds used in a number of industries including textiles, leather, paper, cosmetics, and food. Over 100,000 different dyes have been synthesized, and more than 0.7 million tons are produced annually.^5,6^ Any effluent discharged by the textile industry is a major polluter and source of dyes released into the environment, as dyes are lost to the environment at every step of the process, from manufacture (10–15%) to application (20–30%).^7,8^

**Figure 1:**
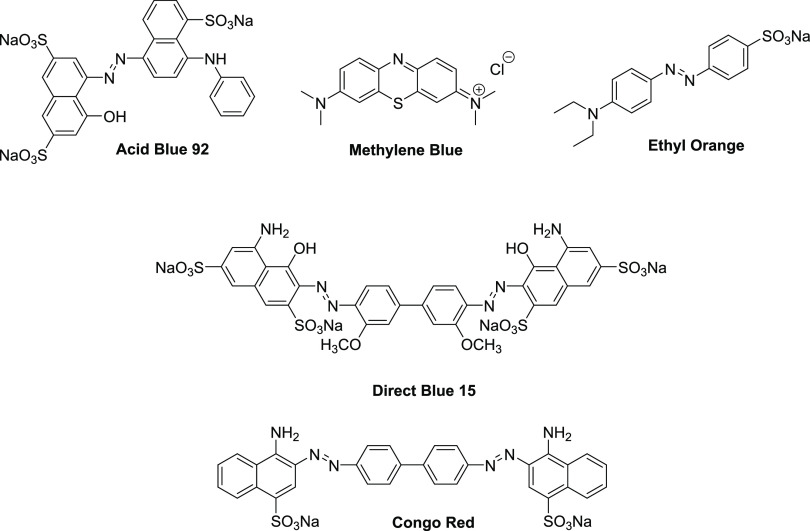
Aromatic organic dye structures investigated within this study.

In this study, we investigate the removal of five aromatic organic compounds as model dye pollutants: Acid Blue 92 (AB), Methylene Blue (MB), Ethyl Orange (EO), Direct Blue 15 (DB), and Congo Red (CR) (Figure 1). The four anionic azobenzene dyes AB92, EO, DB, and CR contain sulfonic groups (1–4, respectively), making them water-soluble and charged once dissolved (i.e., rendering them hard to remove from aqueous solutions).^9^ The cationic thiazine dye MB is used as both a dye and medication, though it is reported as harmful at high concentrations (>2 mg/kg).^10^

Once these dyes enter water bodies and break down, they form toxic and carcinogenic products, including naphthalene and benzamine. They also reduce levels of dissolved oxygen and light penetration due to their colored nature. Lower levels of oxygen and light inhibit the ability of aquatic plants and algae to photosynthesize, which in turn disrupts the aquatic ecosystem and biodiversity. In terms of damage to human health, it has been reported that dyes are carcinogenic and mutagenic as well as are irritants, causing contact dermatitis.^11−13^ It is therefore imperative to remove dyes from water supplies at source.

Methods of dye removal or degradation can be categorized into three main groups: chemical (e.g., oxidation and reduction methods, photochemical degradation, electrochemical degradation, coagulation, ionic separation, and neutralization), physical (adsorption, filtration, precipitation, and reverse osmosis), or biological (sorption or degradation by plants, enzymes, or microbes).^9^ Of these methods, adsorption is the most effective and widely investigated technique for contaminant removal: it is facile to process and possesses high efficiency and high selectivity when compared with degradation techniques.

Currently, activated carbon is the primary adsorbent employed in industry (2280 K tons consumed annually).^14^ However, these materials face challenges regarding recyclability and sustainability and are often disposed of in landfill.^15^ While natural-based (e.g., polysaccharide)^16,17^ alternatives have been well researched for contaminant absorption applications, synthetic porous organic framework materials (POFs) are largely unexplored in the field despite representing a class of materials well suited to purification of water via adsorption. They possess high surfaces areas as well as exhibiting tunability of molecular functionality, physical properties, and surface-aqueous interactions.^18−20^

**Scheme 1:**
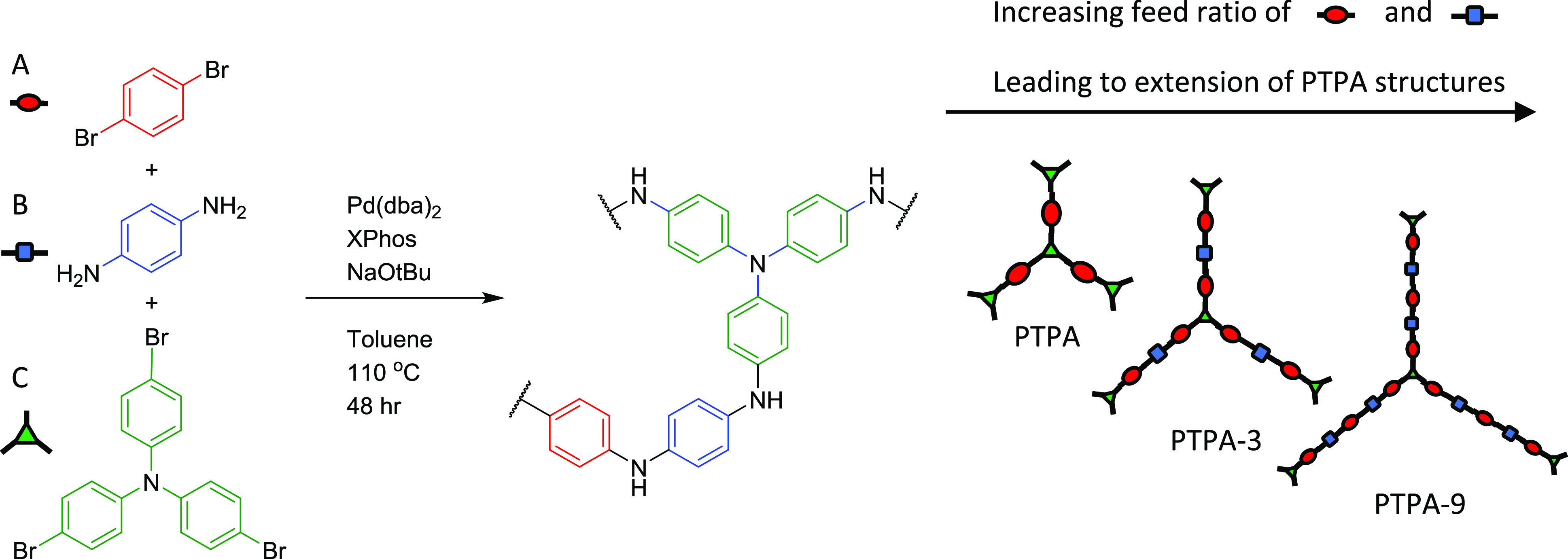
Synthetic Route to **PTPA** and Extended **PTPA 3**–**50**

Here, we present a class of POFs based on cross-linked polyaniline structures^21^ (Scheme 1) for water purification by dye removal. The formed materials are not only able to remove a range of organic dyes from water, but post-synthesis modification (via blending and proof-of-concept device formulation) and variations in adsorption conditions (pH, temperature, and salinity) demonstrate the system’s versatility. Finally, chemical modification of the networks allows targeting of specific organic dyes not adsorbed by the original systems.

## Experimental Section

### Chemicals

Tris(4-bromophenyl)amine (98%), *p*-phenylenediamine (99%), 1,4-dibromobenzene (95%), bis(dibenzylideneacetone)palladium(0) (Pd(dba)_2_), 2-dicyclohexylphosphino-2′,4′,6′-triisopropylbiphenyl (XPhos, 97%), sodium *tert*-butoxide (NaOtBu, 97%), 3,5-dibromobenzoic acid, and all solvents of A.R and C.R. grades were purchased from Merck and used as received. For dye absorbance studies, Acid Blue 92 (40%), Methylene Blue (82%), Congo Red (35%), Ethyl Orange (90%), and Direct Blue 15 (40%) were purchased from Merck and used as received.

### Synthesis

For **PAni**, **PTPA**, and **PTPA**-**3**, **PTPA**-**9**, **PTPA**-**15**, and **PTPA**-**50**, a Schlenk tube was charged with the correct ratios of the monomers and catalysts (see Scheme 1 and Table 1 for ratios and Table S1 for specific feed amounts used). An example is given for **PTPA-3**, where tris(4-bromophenyl)amine (0.25 mmol, 120.5 mg), *p*-phenylenediamine (3 mmol, 324.4 mg), 1,4-dibromobenzene (2.25 mmol, 530.8 mg), Pd(dba)2 ((dba = dibenzylideneacetone) 0.22 mmol, 201.5 mg, 4 mol %), 2-dicyclohexylphosphino-2′,4′,6′-triisopropylbiphenyl (XPhos, 238.4 mg, 0.50 mmol), and sodium *tert*-butoxide (NaOtBu, 384.4 mg, 4 mmol) were added to a Schlenk tube and placed under a nitrogen atmosphere (see Table S1 for the full table of reactant masses added). Anhydrous toluene (50 mL) was added, and the reaction mixture was heated under stirring to 110 °C. After 48 h, TLC analyses were carried out indicating complete consumption of the starting tris(4-bromophenyl)amine. The reaction was cooled to room temperature, and solids were removed by centrifugation. The solids were then subjected to Soxhlet extraction in chloroform, ethanol, methanol, and MQ water (400 mL each for 24 h) and dried for 72 h in a vacuum oven to yield corresponding amine networks as black-blue powders (see Figure S1) with yields of 65–80% (see Table 1).

**Table 1:** Feed Ratios, Yields, Contact Angles (Averaged over a 5 min Period, One Measurement per Second), and Surface Areas for **PAni**, **PTPA**, and Extended **PTPA 3**–**50** (All from Buchwald–Hartwig Cross-Coupling) where A, B, and C Correspond to Starting Materials Shown in Scheme 1

	reactant ratios (molar)						
name	A	B	C	yield (%)	water contact angle (°)	*S*_BET_ (m^2^ g^–1^)	half pore width (Å)
**PTPA**	1.5		1	89	133.3 ± 0.97	171	5.1
**PTPA-3**	12	9	1	98	63.5 ± 0.91	71	37.3
**PTPA-9**	21	18	1	100	79.7 ± 1.12	56	180.0^a^
**PTPA-15**	48	45	1	68	64.1 ± 0.93	74	180.0^a^
**PTPA-50**	153	150	1	72	83.7 ± 1.00	47	180.0^a^
**PAni**	1	1		94	68.7 ± 2.1	18	n/a

a These values represent a maximum of the measurement range, and the materials show pores in the micro- and mesoporous ranges in these instances.

### Characterization and Measurements

Fourier transform infrared (FT-IR) spectra were recorded on a PerkinElmer Spectrum 100 spectrometer, with samples in powder form. Thermogravimetric analysis (TGA) was carried out on a TGA Q500 apparatus in a nitrogen atmosphere (flow rate, 30 mL/min) in the temperature range of 30–800 °C (heating rate, 20 °C/min). Scanning electron microscopic (SEM) images were obtained on a JEOL 5600LV. X-ray diffraction (XRD) patterns were obtained on a Bruker D8 Advance diffractometer (40 kV, 30 mA) using Cu K_α_ radiation (2θ = 5–45°). Nitrogen adsorption/desorption measurements at 77.4 K were performed after degassing the samples on a Schlenk line for 24 h and then under high vacuum at 70 °C for at least 20 h on a Quantachrome Quadrasorb SI-MP apparatus. The specific surface areas were calculated by applying the Brunauer–Emmett–Teller (BET) model to adsorption or desorption branches of the isotherms (N_2_ at 77.4 K) using the QuadraWin 5.05 software package. Analyses of the isotherms by commercialized quenched solid density functional theory (QSDFT)^22^ and Grand canonical Monte Carlo (GCMC)^23^ methodologies were also undertaken using the QuadraWin 5.05 package. The pore size distribution (PSD) profiles of the **PTPA**s were calculated from the adsorption branch of the isotherms with the Grand canonical Monte Carlo (GCMC) approach. ^1^H NMR experiments were performed in D_2_O using Varian VNMR 400 MHz NMR. Contact angle measurements were performed at a temperature of 20 ± 0.5 °C using a drop shape analyzer, DSA100 (KRÜSS), with 10 μL of Milli-Q water (resistivity of 18.2 MΩ·cm) droplets. The image of the drop was recorded for 300 s in 1 s intervals, and at least two repeat measurements per sample were made. UV–vis–NIR spectroscopy measurements were carried out using a Shimadzu UV-2600 spectrometer fitted with an ISR-2600 integrating sphere attachment. Measurements were recorded in 10 mm path length quartz cuvettes.

### Dye Adsorption/Desorption Method

CMP (20 mg) was dispersed in deionized water (pH 7) by sonicating for 5 min. The dispersion was then exposed to the dissolved dye (40 mL) at initial concentrations of 0.0130 mg/mL (EO), 0.0156 mg/mL (AB), 0.0241 mg/mL (DB), and 0.0177 mg/mL (CR). Varying concentrations were used to achieve an absorbance of less than 1 unit, within the linear Beer–Lambert law range. Concentration change was determined over time (24 h) following the decrease in absorbance of selected ƛ_max_ values for EO (475 nm), AB (575 nm), DB (600 nm), and CR (497 nm). The decrease in ƛ_max_ absorbance was correlated to the dye concentration using a concentration curve with an *R*^2^ > 0.99. For the generation of filter absorption units, a Millex syringe filter was used (pore size, 0.8 μm; diameter, 33 mm) and **PTPA-15** was loaded via flowing a dispersion of 10 mg/mL in DI water through the tip. For pH variation studies/desorption of EO, the same procedures were employed (after dye adsorption onto 20 mg of CMP for desorption) with a change of pH (via addition of HCl_(aq)_ or NaOH_(aq)_ to the desired pH) and ƛ_max_ values for EO of 500 nm for pH <4 (no shift in ƛ_max_ values for alkaline pHs was observed; a decrease in ƛ_max_ absorbance under acidic conditions was correlated to the dye concentration using a concentration curve with an *R*^2^ > 0.99).

## Results and Discussion

### Synthesis and Preliminary Characterization

**Figure 2:**
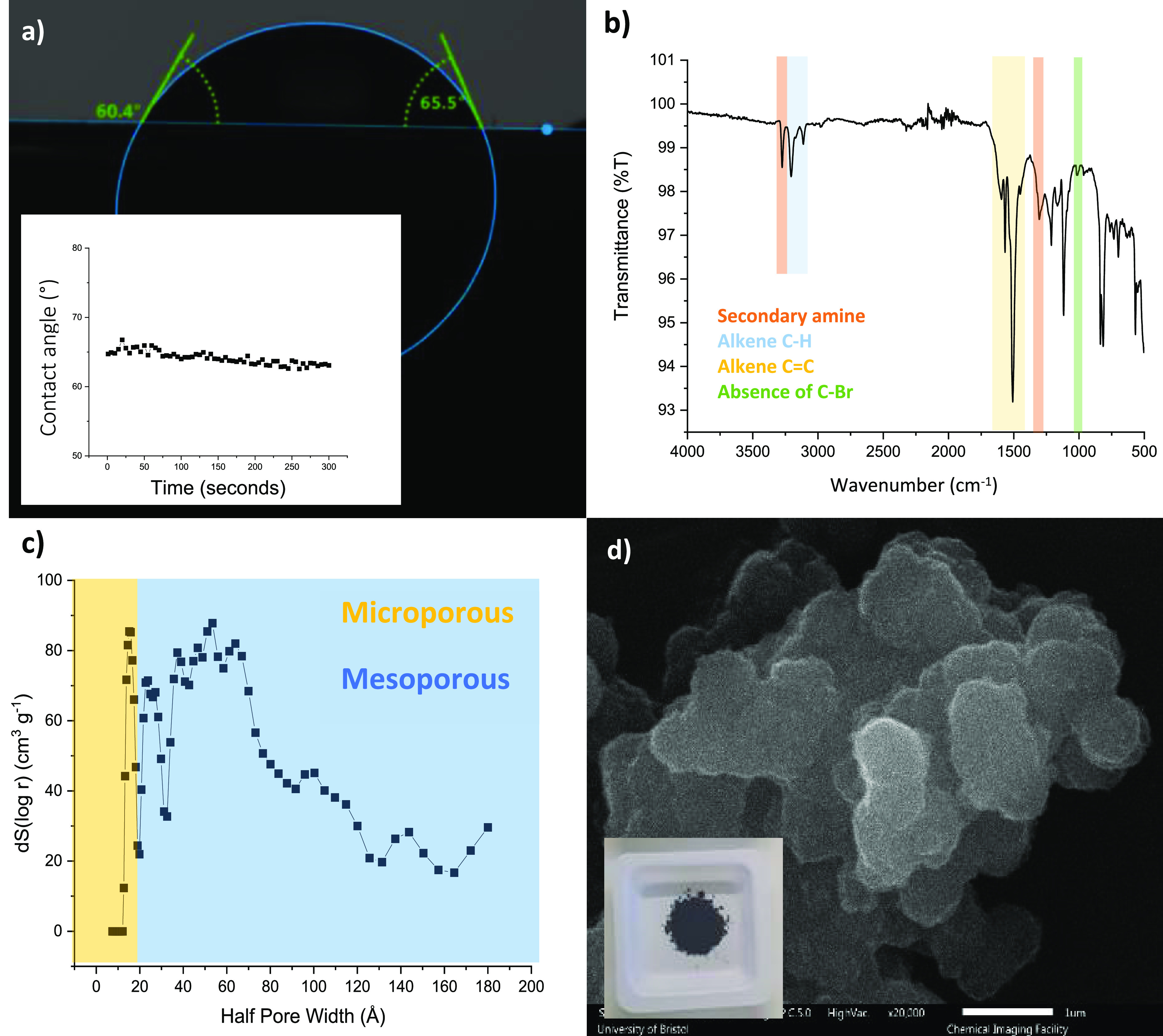
Example characterization data set from extended **PTPA 15** showing (a) contact angle image and measurement over 5 min, (b) FTIR spectrum, (c) DFT analysis showing pore size distribution taken at 77.36 K in a nitrogen atmosphere, and (d) SEM image of the powder product after purification.

The **PTPA** extended networks **PTPA-3**-**50** and the linear **PAni** (Scheme 1 and Table 1) were synthesized using a Buchwald–Hartwig cross-coupling reaction previously reported.^17^ The successful synthesis of cross-linked networks was confirmed using several techniques (see Figure 2a–d for example data for **PTPA-15**). FT-IR spectroscopy demonstrated the disappearance of the carbon–bromine bond stretching vibration at 1004 cm^–1^ associated with the starting material (Figure 2b and Figure S2). Furthermore, the appearance of the secondary amine stretching vibration at 1305 cm^–1^ also suggested successful synthesis. UV–vis solid-state analysis of the networks revealed absorbances at 342 and 660 nm, characteristic of the π–π* transition of benzenoid and quinoid rings, respectively (Figure S3).^12^ Interestingly, it was observed that the relative intensity of the quinoid-based transition, with respect to the benzenoid, decreased as the linker length increased. Thermal analysis of the networks **PTPA** and **PTPA-3**-**50** revealed degradation temperatures in the range of 232–270 °C (Table S2 and Figure S4); no direct correlation between degradation temperature and linker length was observed, although a significant increase in thermal stability with respect to **PAni** (193 °C) was observed. No thermal transitions were apparent at lower temperatures, as expected in highly cross-linked networks. XRD analysis of the networks revealed broad amorphous features centered at 2θ = 12.5°, similar to earlier characterization of these and related materials (Figure S5). It is noted that the synthesized **PAni** was in the emeraldine base (EB) state.

To assess the suitability of the networks as adsorbents, surfaces areas were determined by BET surface area analysis. Analysis revealed a sharp decrease in surface area when extending networks (see Table 1) and an increase in the overall half pore width toward the mesoporous region (see Figure S6). As the mesoporous content of the networks increased, the networks tended toward the extended structure of the linear **PAni**, with consequent more favorable water interactions (i.e., more hydrophilic). This trend was first observed qualitatively by simple water dispersion tests: the extended networks retained superior dispersibility with respect to **PTPA** (Figure S7). This trend was confirmed by contact angle measurements. The contact angle decreased from 133.3° for **PTPA** (hydrophobic) to a range of 63.5–83.7° (hydrophilic) for the extended networks **PTPA 3**–**50** and **PAni** (see Figure 2a for **PTPA-15** and Table 1 and Figure S8).^24,25^ Interestingly, the extended networks of **PTPA 3**–**50** do not follow a uniform trend of decreasing contact angle toward that of **PAni** (increase seen for **PTPA-9** and **PTPA-50**). This data suggests that, while the overall decrease in contact angle may be due to polyaniline-like linkers, the variations of contact angle in the extended region are likely caused by variations in the topology of the surfaces (corroborated by pore size distributions, see Figure 2c, SEM analysis in Figure 2d, and dye uptake kinetic studies discussed later).^26,27^

### Dye Adsorption Analysis

**Figure 3:**
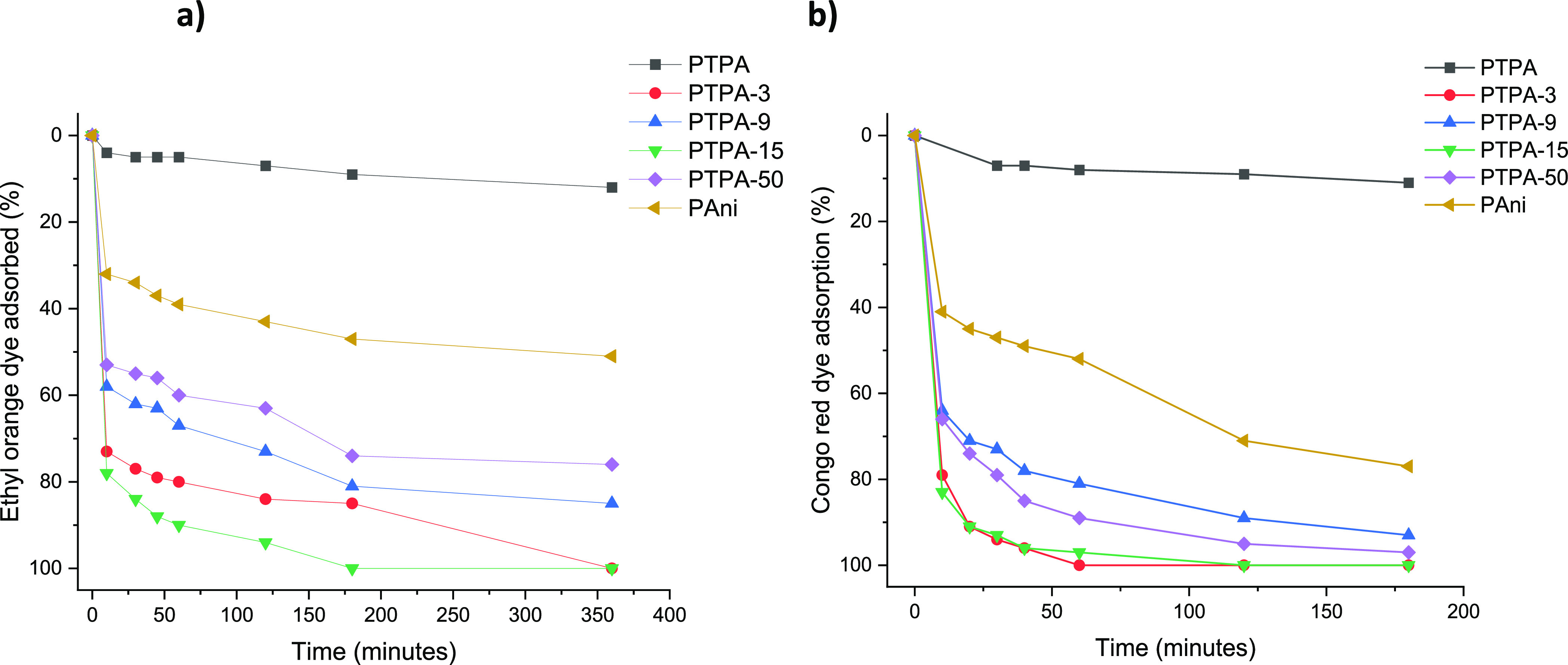
Comparison of the networks **PTPA**, **PTPA 3**–**50**, and **PAni** as organic dye adsorbents: (a) EO adsorption (%) (initial concentrations of 0.0130 mg/mL) and (b) CR adsorption (%) (initial concentrations of 0.0177 mg/mL) both at pH 7, over 360 min at 24 °C determined by UV–vis.

EO was initially selected to assess the suitability of the polymeric networks as an organic dye adsorbent (from aqueous systems at pH 7, see the Experimental Section for details). It was found that the 15-extension **PTPA-15** performed as the most effective dye scavenger with over 75% of dye absorbed within 5 min at a rate of 0.041 mg/min when using 20 mg of the adsorbent (Figure 3 and Table 2 and Figures S10–S12). It is worth noting that the extensions performed significantly better than the parent **PTPA** and **PAni** materials, with adsorption rates of 0.002 and 0.017 mg/min, respectively. For **PTPA**, it is suggested that the lower adsorption rate is due to the poor dispersibility of the networks in the aqueous environment, leading to a lack of contact with the dye (see contact angle data in Table 1 and discussion above). For **PAni**, it is suggested that the lack of porosity does not allow successful contact and dye entrapment. This aspect was illustrated in other studies, where porosity and uptake of **PAni** systems were enhanced by composite formation with porous materials.^28^

**Table 2:** Maximum Rate of Adsorption (*K*_a_ in mg/min Derived from the First 5 min of Absorption) and Maximum Dye Absorbed (Abs_max_ in mg/g, Derived from Solution Saturation) of Anionic Azobenzene Dyes by **PTPA**-Derived Networks

	Congo Red		Ethyl Orange		Acid Blue		Direct Blue	
name	*K*_a_ (mg/min)	Abs_max_(mg/g)	*K*_a_ (mg/min)	Abs_max_(mg/g)	*K*_a_ (mg/min)	Abs_max_(mg/g)	*K*_a_ (mg/min)	Abs_max_(mg/g)
**PTPA**	0.008	13	0.002	17	0	20	0	18
**PTPA-3**	0.060	60	0.038	49	0.046	74	0.053	116
**PTPA-9**	0.048	50	0.030	49	0.008	43	0.035	57
**PTPA-15**	0.063	105	0.041	51	0.038	74	0.053	111
**PTPA-50**	0.050	78	0.028	42	0.014	53	0.045	118
**PAni**	0.031	31	0.017	25	0.021	41	0.024	55

**Figure 4:**
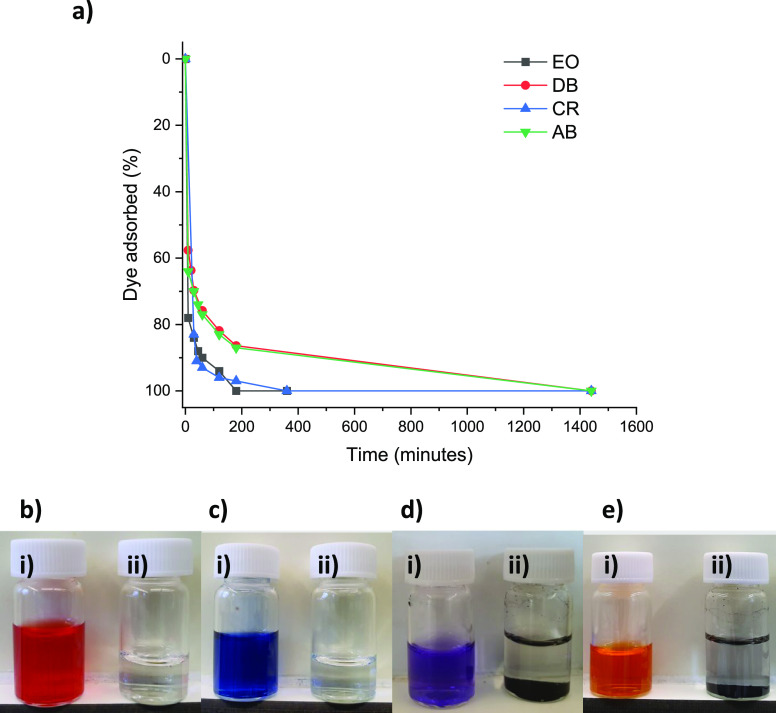
Adsorption of four organic dyes by **PTPA-15** determined by (a) UV–vis spectroscopy and visually, where panel (b) is CR, panel (c) is DB where (i) is before network addition and (ii) is after network addition and filtration, panel (d) is AB, and panel (e) is EO where (i) is before network addition and (ii) is after network addition but before filtration (to demonstrate visually the impact of adsorption) over 1500 min (at 24 °C, pH 7, for initial dye concentrations, see the Experimental Section).

Subsequent organic dyes tested on the networks included the anionic dyes AB, CR, and DB and the cationic dye MB. For each of the anionic dyes (Figure 1), increased removal capabilities were demonstrated by the extended networks over **PTPA** and **PAni**, with **PTPA-15** proving to be the most efficient with respect to dye adsorption over time as well as the range of dyes absorbed (all anionic dyes removed within 1400 min, monitored by UV–vis; Figure 4). The increased removal rate of both CR and EO with respect to DB and AB is attributed to the latter dyes’ solubility being a factor of 10 greater than that of both the former. Furthermore, analysis of rate of absorption kinetics revealed good fits (*R*^2^ > 0.8, see Table S3) for extensions 9, 15, and 50 within the pseudo-first-order absorption model for all dye absorptions.^29^ Interestingly, **PTPA** showed low fitting values across individual order models and **PTPA-3** showed better fitting for pseudo-second-order absorptions, potentially due to the surface topography (see Table 1), implying that the absorption process was enhanced by chemisorption.^30^ The maximum absorption observed (where the solutions were saturated with dyes until no more absorption was observed by UV–vis measurements) reported in Table 2 shows dramatically increased uptakes when compared to non-synthetic dye-absorbent systems^31,32^ (e.g., CR Abs_max_ of 1 mg/g for plant material-derived systems vs 105 mg/g for **PTPA-15**) and competitive uptakes compared to activated carbon black (CR Abs_max_ of 100 mg/g, see Table S4 for full comparative figures).^33−36^ It is worth noting that the removal of the cationic dye MB was not achieved with these amino-based motifs (see discussion below).

Model binding studies were undertaken to understand the nature of the interactions of the organic dyes with the **PTPA** networks. Here, dilutions of CR (other dyes were not studied as signal dependency on the concentration was too low) in the presence of diphenyl amine (as the linker model) were analyzed using ^1^H NMR in DMSO-*d*_6_ (to ensure full solvation of both the dye and linker model, see Figure S13). Eight dilutions were made with a total combined concentration of 8 mg/mL for each of the dye and linker (0:8, 1:7, 2:6, 3:5, 4:4, 5:3, 6:2, 7:1, and 8:0 of dye to linker, respectively). The studies revealed changes to lower chemical shifts of the amidic protons of the CR with increasing linker concentration (from 7.685 to 7.675 ppm), indicating an increase in hydrogen bonding. However, the limited changes observed in aromatic resonances indicate a lack of π–π stacking interactions between the dye and the linker, at least for this model system. These investigations further confirmed that the surface area and porosity are the main factors accounting for the difference between the dye adsorption properties of the extended **PTPA** networks and **PAni**. The better performance of **PTPA-15** when compared with other extended networks is linked to the increased propensity for dispersion as demonstrated by contact angle analysis (see Figure S14).

### Variation of Aqueous Conditions of Dye Adsorption Studies

**PTPA-15** was selected as the most versatile of the adsorbent materials to assess the effect of aqueous conditions on dye adsorption capacities of the extended networks (see Figures 3 and 4), with the salt content, temperature, and pH varied. First, the adsorption of CR was undertaken in saline conditions (35 mg/mL) to mimic industry relevant conditions. It was found that the addition of sodium chloride increased dye uptake (Figure S14), with no CR dye detectable by UV–vis investigation after 10 min (in comparison to 360 min with no salt added). Although salt addition is a reported way of precipitating CR from aqueous environments (due to enhanced π–π stacking and lowered solubility^37^), the use of the network as an adsorbent compared with simple precipitation using salt allows for removal of dyes at a far superior rate (minutes vs weeks) under mimicked conditions.^38^

Elevated temperature adsorption investigations of CR with **PTPA-15** were also conducted to mimic conditions encountered in industry. Adsorption was monitored in a water bath set at 80 °C, while all other conditions (including solvent volumes, dye, and network amounts) were kept constant. It was again found that elevated temperatures aided adsorption, with 100% of CR adsorbed within 10 min (Figure S15). While the rapid increase in dye absorption suggests a change from a pseudo-first-order to a second-order mechanism (see Table S3), it is likely that simple increased diffusion of both the dye and CMP leads to increased rates.^27,28^

**Figure 5:**
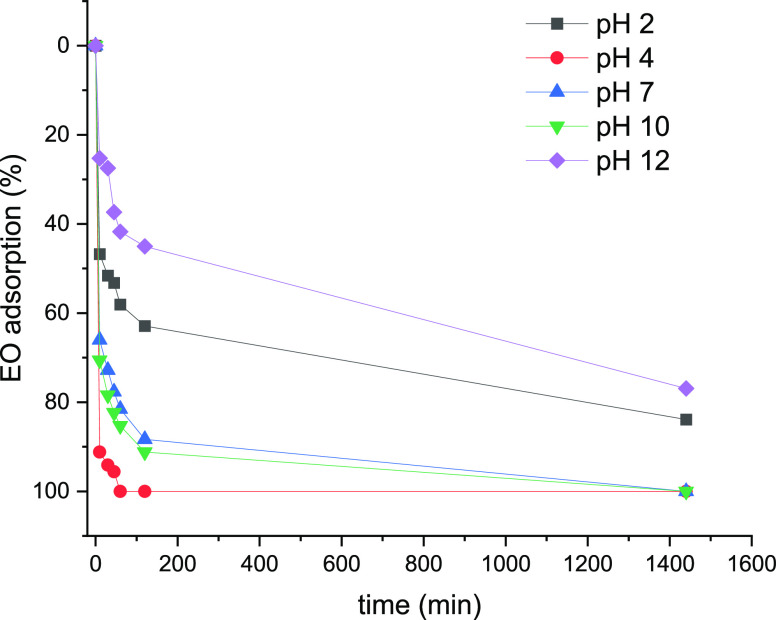
EO adsorption (initial concentrations, 0.0130 mg/mL) by **PTPA**-**15** as a function of pH, at 24 °C over 1500 min (n.b. ƛ_max_ values 500 nm for pH < 4, 480 nm for pH > 7).

EO was then selected as a model dye to study the effect of pH on the adsorption capabilities (CR was incompatible with such studies as its solubility is greatly decreased by variations in pH).^39^ It was found that very low or high pH (2 and 12) reduced the uptake capacity of the dye to the network. Moderate changes in pH toward the p*K*_a_ of the dye (4.34) gave increased uptake capacity (especially pH 4; Figure 5). It is proposed that the quinoidal state of the protonated EO allows for a better interaction with the networks at pH 4 (see the solid-state UV–vis spectra of the networks in Figure S3, showing dominance of quinoidal states in the extended networks). At lower pH values (2), the protonations of both sulfonate and amine groups of the dye and network, respectively, cause less attraction resulting in disruption of absorption (72% adsorbed after 1400 min). At higher pH values (12), the dye is fully deprotonated, hence loses the ability to hydrogen bond with the extended network, and therefore is not adsorbed efficiently (61% only after 1400 min). The theorized interactions of EO and the polyanaline-like networks are represented in Figure S16.

### Modification of the Adsorbent Systems

Three additional modifications and investigations were undertaken to demonstrate the versatility of the extended networks as dye-absorbing materials and included preparing blended composites, exploring the dependence on pH of the environment, and changing the molecular architecture and function to target specific dyes for removal.

**Figure 6:**
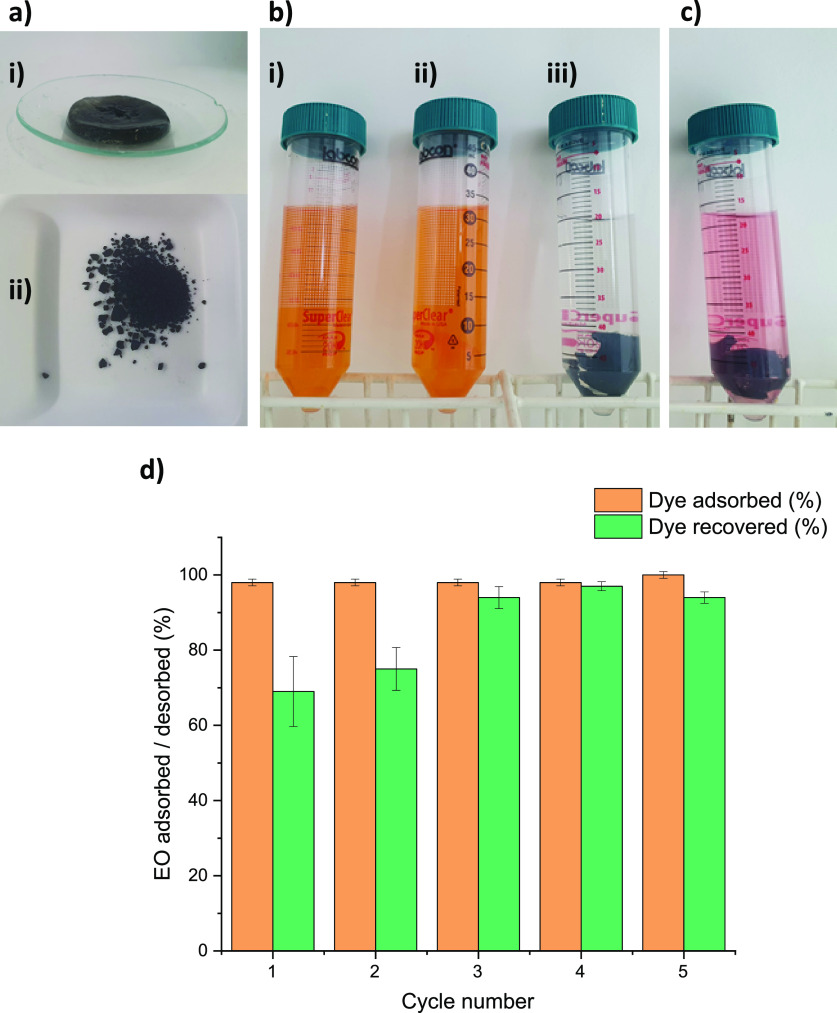
(a) **PTPA-15** in (i) powder and (ii) blended gel form; (b) EO in (i) water/no gel, (ii) the presence of a pure gel insert (control with no CMP addition) after 24 h, and (iii) fully absorbed by the gel insert with 10 wt % **PTPA-15** after 24 h; (c) release of EO after addition of HCl to the gel containing 10 wt % **PTPA-15**; and (d) recyclability of the composite system as monitored by UV–vis spectroscopy.

First, entrapment of the extended networks within a hydrogel was undertaken to improve manipulation of the formed blends (with respect to the powder-like form of the extended networks) after dye absorption. This approach ensured that a single solid mass could be removed from the aqueous solution after absorbance, rather than filtration being necessary, as shown in Figure 6. Hydrogels were formed from the cross-linking of polyvinyl alcohol (PVA) with terephthalaldehyde (TPA) in the presence of 37% HCl_(aq)_. Gels were either prepared with a 10 wt % loading of extended **PTPA-15** or as control with no **PTPA-15**. It is worth noting that higher wt % **PTPA-15** loading caused a failure in cross-linking, leading to unstable gels. After formation, gels were washed with water until neutral pH was achieved.

Despite control gels placed in the EO solution not absorbing any dye (Figure 6b, (ii), no decrease in the EO concentration was detected by UV–vis), complete absorption of EO (0.0130 mg/mL) was achieved after 24 h by the prepared composite CMP-containing gels (Figure 6b, (iii)). The practical advantage of our approach here is the facile removal of the gel after dye removal without the need for any filtration equipment (Figure 6b, (iii)). To test for possible contamination of the systems or CMP degradation, the experiment was rerun in D_2_O and the supernatant of the fifth cycle was analyzed using ^1^H NMR analysis, revealing no impurities or degraded product (Figure S17).

In addition, to explore the ability to recycle the loaded CMPs or their gel composites, we explored the use of facile pH changes to release the absorbed EO dye. For the gel/**PTPA-15** blend (Figure 6c,d), addition of 1 M HCl_(aq)_ (to achieve pH 2) led to 100% EO release over 30 min (Figure 6c). The use of pH to control absorbance and release for the parent powdered **PTPA-15** was furthermore demonstrated in real time (see Videos S1 and S2, respectively, in the Supporting Information). **PTPA-15** powder was loaded onto a Millex syringe filter, and a 0.013 mg/mL EO stock solution was filtered through to show immediate absorption. The release of this absorbed EO dye was achieved in real time over 30 s by treatment with a HCl solution at pH = 2.

These experiments clearly showed the versatility of the **PTPA-15** in either gel form (Figure 6d, pH treatment of the gel composite and recycled five times) or as a powdered insert (Videos S1 and S2) to absorb and release dyes (with comparable efficiencies to well-researched polysaccharide systems),^40^ showing the significant potential of these materials for real-life applications.

**Figure 7:**
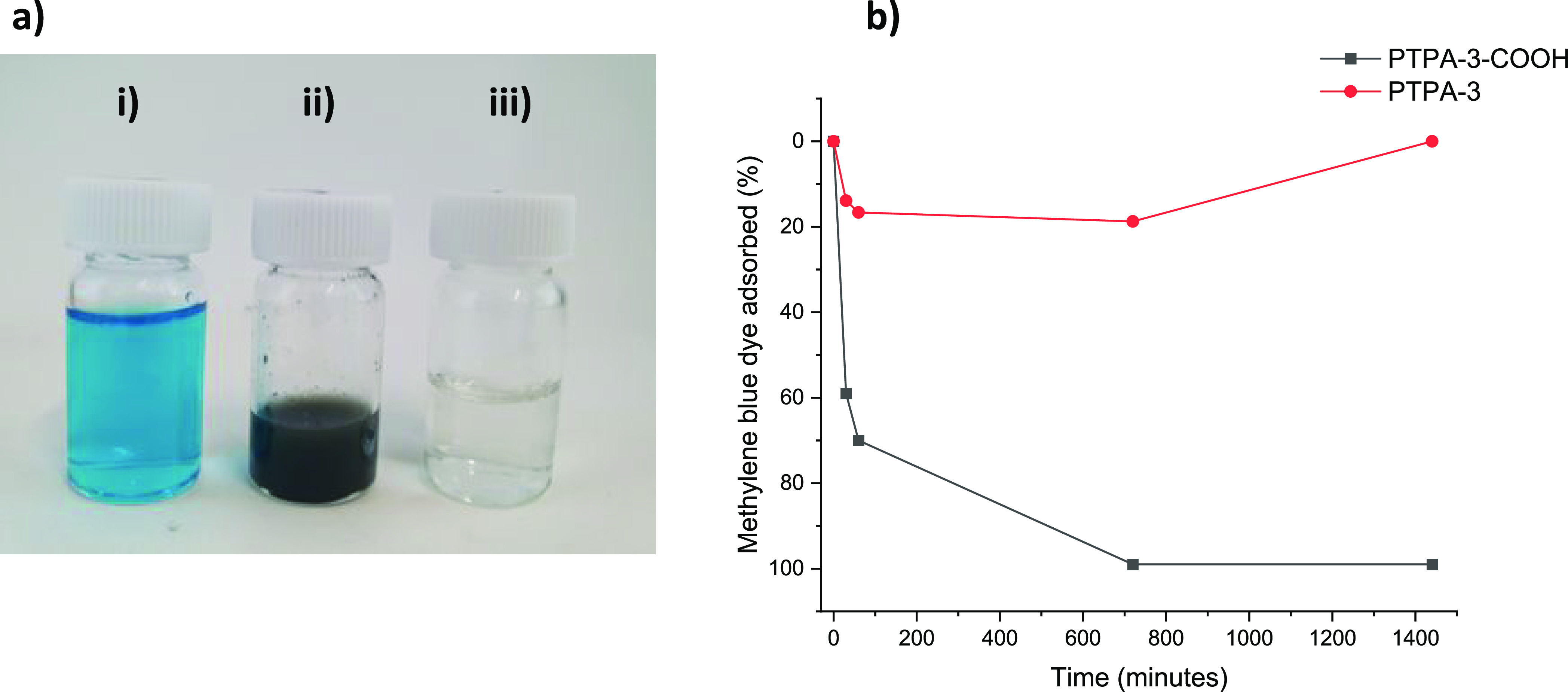
(a) MB adsorption from aqueous solution via addition of functionalized network **PTPA-COOH** where (i) is MB in aqueous solution (0.02 mg/mL), (ii) is MB and **PTPA-3-COOH** (20 mg/mL) in contact in aqueous solution, and (iii) is the solution after filtration after 1400 min of contact with the network and (b) MB adsorption followed by time-dependent UV–vis spectroscopy for **PTPA-3** and **PTPA-3-COOH**.

Finally, structural modifications of the extended networks were undertaken to target the cationic dye MB (not absorbed by networks **PTPA** and **PTPA 3**–**50**). Only modifications of **PTPA-3** (selected as the best performing network) were undertaken. Here, the extended network formed from Buchwald–Hartwig cross-coupling reactions with 3,5-dibromobenzoic acid (rather than 1,4-dibromo phenyl units, see Scheme S1 for a complete reaction pathway) gave the extended structure functionalized with carboxylic acid moieties throughout (**PTPA-3-COOH**). Successful synthesis was confirmed by UV–vis and FTIR spectroscopy and surface area analysis (as for the extended networks, see Figures S2–S5 and S18). Although the functionalized network showed a decrease in surface area (37 m^2^/g), increased water dispersibility was immediately visible (Figure S7) and confirmed by contact angle measurements (69.1 ± 2.3°). Successful removal of the MB dye from water after 700 min was confirmed visually (see Figure 7a, (iii)) and by UV–vis measurements (Figure 7b).

Interestingly, the functionalized **PTPA-3-COOH** was not able to absorb any of the investigated anionic dyes. However, the formation of the anionic network as well as successful removal of MB demonstrates the versatility of these networks in regard to their tunability and synthetic modification for targeted contaminant adsorption.

## Conclusions

In the search for simple and low-cost solutions to produce safe and clean drinking water, the use of heteroatom-containing conjugated microporous polymers was explored. Although such materials are often used in gas-capture settings (especially CO_2_ capture), we have demonstrated here the formation of extended **PTPA** cross-linked networks and their suitability as water purification motifs. Investigation into the networks’ abilities to remove pollutant organic dyes from aqueous systems has enabled initial optimization of the systems, leading to attractive performance and complete removal of cationic dyes in less than 10 min under conditions similar to those found in the dying industry. Further modifications, including blending and composite formation and their recyclability, showed the promise of these materials and approach for simple, low-cost, real-life applications. In addition, we have also shown the versatility of our approach, where simple structural modifications could be introduced to remove anionic dyes in a targeted fashion. Future modifications could include the addition of specific metal-coordinating moieties throughout the framework structure, thus potentially enabling the removal of highly toxic heavy metals such as Hg, Cd, or As. We therefore envisage that these materials will find wide application in low-cost filtration units for use in the informal dye industry and for water purification in rural settings in developing nations with contaminated water sources.

## Supporting Information Available

Figures S1–S19: FTIR, TGA, XRD, BET, contact angle, SEM, UV–vis, adsorption, and NMR data; Tables S1–S4: composition and kinetic data; and Scheme S1: synthetic route to **PTPA-3-COOH** (PDF)
Video S1: The use of pH to control the absorbance for the parent powdered **PTPA-15** (MOV)
Video S2: The use of pH to control the release for the parent powdered **PTPA-15** (MP4)

## Supplementary Material

ap2c01718_si_001.pdf

ap2c01718_si_002.mov

## References

[ref1] United Nations, Transforming Our World: the 2030 Agenda for Sustainable Development, 2015, https://sdgs.un.org/2030agenda, accessed (30/08/2022).

[ref2] UNICEF, WHO, 1 in 3 people globally do not have access to safe drinking water, 2019, https://www.who.int/news/item/18-06-2019-1-in-3-people-globally-do-not-have-access-to-safe-drinking-water-unicef-who, accessed (29/05/2022).

[ref3] UN Development Programme, Building equality on the roof of the world, 2019, https://undp.medium.com/building-equality-on-the-roof-of-the-world-39885f5e35f4, accessed (14/05/2022)

[ref4] Ismail A. F.; Goh P. S.; Carbon-based polymer nanocomposites for environmental and energy applications*;* Elsevier: Amsterdam, Netherlands, 2018.

[ref5] CNN, Regan H., Asian rivers are turning black. And our colorful closets are to blame, 2020, https://edition.cnn.com/style/article/dyeing-pollution-fashion-intl-hnk-dst-sept/index.html, accessed (28/04/2022)

[ref6] Jawaid M.; Kahn M. M., Polymer-based nanocomposites for energy and environmental applications. Elsevier: Oxford, United Kingdom 2018.

[ref7] Singh N. B.; Susan A. B. H.; Polymer nanocomposites for water treatments. in Polymer-based Nanocomposites for Energy and Environmental Applications (eds. Jawaid M.; Khan M. M.) 569–595 (Woodhead Publishing, 2018). doi:10.1016/B978-0-08-102262-7.00021-0.

[ref8] Bhavsar S.; Dudhagara P.; Tank S. R software package based statistical optimization of process components to simultaneously enhance the bacterial growth, laccase production and textile dye decolorization with cytotoxicity study. PLoS One 2018, 13, e0195795 10.1371/journal.pone.0195795. 29718934 PMC5931462

[ref9] Szyguła A.; Guibal E.; Palacín M. A.; Montserrat R.; Sastre A. M. J. Environ. Manage. 2009, 90, 2979–2986. 10.1016/j.jenvman.2009.04.002. 19467769

[ref10] Ginimuge P. R.; Jyothi S. D. J. Anaesthesiol Clin. Pharmacol. 2010, 26, 517–520. 10.4103/0970-9185.74599. 21547182 PMC3087269

[ref11] Chatha S. A. S.; Asgher M.; Iqbal H. M. N. Enzyme-based solutions for textile processing and dye contaminant biodegradation—a review. Environ. Sci. Pollut. Res. 2017, 24, 14005–14018. 10.1007/s11356-017-8998-1. 28401390

[ref12] Kumar L.; Bharadvaja N. Microorganisms: A remedial source for dye pollution. In Removal of Toxic Pollutants Through Microbiological and Tertiary Treatment (ed. Shah M. P.) 309–333 (Elsevier, 2020). Doi:10.1016/B978-0-12-821014-7.00012-5.

[ref13] Kumar P.; Agnihotri R.; Wasewar K.; Uslu H.; Yoo C. Status of adsorptive removal of dye from textile industry effluent. Desalin. Water Treat. 2012, 50, 226–244. 10.1080/19443994.2012.719472.

[ref14] Global Trade, Previously Driven by the Growth of the Chemical Industry and Construction, the Global Activated Carbon Market to Struggle with the Pandemic, 2020, https://www.globaltrademag.com/previously-driven-by-the-growth-of-the-chemical-industry-and-construction-the-global-activated-carbon-market-to-struggle-with-the-pandemic/, accessed (05/06/2022)

[ref15] Song Z.; Chen L.; Hu J.; Richards R. NiO111 nanosheets as efficient and recyclable adsorbents for dye pollutant removal from wastewater. Nanotechnology 2009, 20, 275707 10.1088/0957-4484/20/27/275707. 19531863

[ref16] Zeng S.; Long J.; Sun J.; Wang G.; Zhou L. A review on peach gum polysaccharide: Hydrolysis, structure, properties and applications. Carbohydr. Polym. 2022, 279, 119015 10.1016/j.carbpol.2021.119015. 34980358

[ref17] Song Y.; Tan J.; Wang G.; Zhou L. Superior amine-rich gel adsorbent from peach gum polysaccharide for highly efficient removal of anionic dyes. Carbohydr. Polym. 2018, 199, 178–185. 10.1016/j.carbpol.2018.07.010. 30143118

[ref18] Lee J.-S. M.; Cooper A. I. Advances in Conjugated Microporous Polymers. Chem. Rev. 2020, 120, 2171–2214. 10.1021/acs.chemrev.9b00399. 31990527 PMC7145355

[ref19] Chen J.; Qiu T.; Yan W.; Faul C. F. J. Exploiting Hansen solubility parameters to tune porosity and function in conjugated microporous polymers. J. Mater. Chem. A 2020, 8, 22657–22665. 10.1039/D0TA05563H.

[ref20] Zhou Y.-B.; Zhan Z.-P. Conjugated Microporous Polymers for Heterogeneous Catalysis. Chem. – Asian J. 2018, 13, 9–19. 10.1002/asia.201701107. 29045042

[ref21] Liao Y.; Weber J.; Faul C. F. J. Conjugated microporous polytriphenylamine networks. Chem. Commun. 2014, 50, 8002–8005. 10.1039/C4CC03026E. 24915169

[ref22] Ravikovitch P. I.; Neimark A. V. Langmuir 2006, 22, 11171 10.1021/la0616146. 17154599

[ref23] AS. Samios S.; Stubos A. K.; Kanellopoulos N. K.; Cracknell R. F.; Papadopoulos G. K.; Nicholson D. Langmuir 1997, 13, 2795 10.1021/la962111a.

[ref24] Ding H.; Zhu C.; Zhou Z.; Wan M.; Wei Y. Macromol. Rapid Commun. 2006, 27, 1029–1034. 10.1002/marc.200600143.

[ref25] Law K.-Y. Definitions for Hydrophilicity, Hydrophobicity, and Superhydrophobicity: Getting the Basics Right. J. Phys. Chem. Lett. 2014, 5, 686–688. 10.1021/jz402762h. 26270837

[ref26] Krainer S.; Hirn U. Contact angle measurement on porous substrates: Effect of liquid absorption and drop size. Colloids Surf., A 2021, 619, 126503 10.1016/j.colsurfa.2021.126503.

[ref27] Wang X.; Chen B.; Dong W.; Zhang X.; Li Z.; Xiang Y.; Chen H. Hydrophilicity-Controlled Conjugated Microporous Polymers for Enhanced Visible-Light-Driven Photocatalytic H_2_ Evolution. Macromol. Rapid Commun. 2019, 1800494 10.1002/marc.201800494. 30556197

[ref28] Shen J.; Shahid S.; Amura I.; Sarihan A.; Tian M.; Emanuelsson E. A. C. Enhanced adsorption of cationic and anionic dyes from aqueous solutions by polyacid doped polyaniline. Synth Metals 2018, 245, 151–159. 10.1016/j.synthmet.2018.08.015.

[ref29] Kajjumba G. W.; Emik S.; Öngen A.; Özcan A.H.; Aydın S.; in Edebali S (ed.). Advanced Sorption Process Applications, IntechOpen, London, 2019. 10.5772/intechopen.75857

[ref30] Ahmad R.; Mirza A. Synthesis of guar gum/bentonite a novel bionanocomposite: isotherms, kinetics and thermodynamic studies for the removal of Pb (II) and crystal violet dye. J. Mol. Liq. 2018, 249, 805–814. 10.1016/j.molliq.2017.11.082.

[ref31] Wekoye J. N.; Wanyonyi W. C.; Wangila P.T.; Tonui M. K. Kinetic and equilibrium studies of congo red dye adsorption on cabbage waste powder. Environ. Chem. Ecotoxicol. 2020, 2, 24–31. 10.1016/j.enceco.2020.01.004.

[ref32] Wanyonyi W. C.; Onyari J. M.; Shiundu P. M. Adsorption of Congo Red Dye from Aqueous Solutions Using Roots of Eichhornia Crassipes: Kinetic and Equilibrium Studies. Energy Proc. 2014, 50, 862–869. 10.1016/j.egypro.2014.06.105.

[ref33] Lafi R.; Montasser I.; Hafiane A. Adsorption of congo red dye from aqueous solutions by prepared activated carbon with oxygen-containing functional groups and its regeneration. Adsorpt. Sci. Technol. 2019, 37, 160–181. 10.1177/0263617418819227.

[ref34] Ali F.; Ali N.; Bibi I.; Said A.; Nawaz S.; Ali Z.; Salman S. M.; Iqbal H. M. N.; Bilal M. Adsorption isotherm, kinetics and thermodynamic of acid blue and basic blue dyes onto activated charcoal. Case Stud. Chem. Environ. Eng. 2020, 2, 100040 10.1016/j.cscee.2020.100040.

[ref35] Prola L. D. T.; Machado F. M.; Bergmann C. P.; de Souza F. E.; Gally C. R.; Lima E. C.; Adebayo M. A.; Dias S. L. P.; Calvete T. Adsorption of Direct Blue 53 dye from aqueous solutions by multi-walled carbon nanotubes and activated carbon. J. Environ. Manage. 2013, 130, 166–175. 10.1016/j.jenvman.2013.09.003. 24076517

[ref36] Dutta S.; Gupta B.; Srivastava S. K.; Gupta A. K. Recent advances on the removal of dyes from wastewater using various adsorbents: a critical review. Mater. Adv. 2021, 2, 4497–4531. 10.1039/D1MA00354B.

[ref37] Skowronek M.; Stopa B.; Konieczny L.; Rybarska J.; Piekarska B.; Szneler E.; Bakalarski G.; Roterman I. Biopolymers 1998, 46, 267–281. 10.1002/(SICI)1097-0282(19981015)46:5<267::AID-BIP1>3.0.CO;2-N.

[ref38] Bancroft J. D.; Gamble M.; Theory and Practice of Histological Techniques, Elsevier, 2007.

[ref39] Bayliss W. M. The properties of colloidal systems. I.-The osmotic pressure of congo-red and of some other dyes. Proc. R. Soc. Lond. B. 1909, 81, 269–286. 10.1098/rspb.1909.0023.

[ref40] Zeng S.; Tan J.; Xu X.; Huang X.; Zhou L. Facile synthesis of amphiphilic peach gum polysaccharide as a robust host for efficient encapsulation of methylene blue and methyl orange dyes from water. Int. J. Biol. Macromol. 2020, 154, 974–980. 10.1016/j.ijbiomac.2020.03.151. 32198040

