# Supplementary material for: Controlled Removal of Organic Dyes from Aqueous Systems Using Porous Cross-Linked Conjugated Polyanilines
Source: ACS Appl Polym Mater. 2022 Dec 19;5(1):662–71. doi: 10.1021/acsapm.2c01718 (PMC9841504; doi:10.1021/acsapm.2c01718)
Supplement: Supplementary file 1 — ap2c01718_si_001.pdf [file ap2c01718_si_001.pdf]

Supporting Information

Controlled removal of organic dyes from aqueous systems using porous crosslinked conjugated polyanilines

Julia C. Maxwell, Benjamin C. Baker, Charl F. J. Faul*

School of Chemistry, University of Bristol, Cantock's Close, Bristol, BS8 1TS, UK
Corresponding author email; Charl.Faul@bristol.ac.uk

Figure S1: Powders of left to right	2
Table S1: Feed ratios for formation of the extended networks	2
Figure S2: FTIR of extended networks	3
Figure S3: Solid state UV-vis of extended networks	3
Table S2: Decomposition temperatures as determined by TGA	4
Figure S4: TGA of the extended networks	4
Figure S5: XRD analysis of the extended networks	5
Figure 6a: Nitrogen BET isotherms of the extended networks collected at 273 K	5
Figure 6b: DFT pore size distribution of the extended networks	6
Figure S7: Aqueous dispersions of extended networks	6
Figure S8a: Contact angle images of the extended networks	7
Figure S8b: Contact angle measurements of the extended networks	7
Figure S9: SEM images of the extended networks	8
Figure S10a: UV-vis spectra showing the adsorption of congo red dye	8
Figure S10b: UV-vis spectra showing the adsorption of ethyl orange dye	9
Figure S10c: UV-vis spectra showing the adsorption of acid blue dye	9
Figure S10d: UV-vis spectra showing the adsorption of direct blue dye	10
Figure S11a: Adsorption of congo red dye over time by extended networks	10
Figure S11b: Adsorption of ethyl orange dye over time by extended networks	11
Figure S11c: Adsorption of acid blue dye over time by extended networks	11
Figure S11d: Adsorption of direct blue dye over time by extended networks	12
Figure S12: Direct comparison of the dye adsorption performance of networks	12
Table S3: Coefficients of correlation (R^2)	13
Table S4: Absorption capacities of Activated carbon of the dyes of interest	13
Figure S13a-c: ^{1}H NMR binding studies	14-16
Figure S14; CR dye absorption as a function of contact angle	17
Figure S15. CR adsorption by PTPA-15 over varying aqueous conditions	17
Figure S16; Theorised interactions between the EO dye and Polyanaline like networks.	18
Figure S17: ^{1}H NMR analysis of dye desorption from gel/PTPA-15 blend	18
Scheme S1: Synthetic route to PTPA-3-COOH	19
Figure S18: Characterisation of PTPA-3-COOH	20
Figure S19: Adsorption of methylene blue dye over time by PTPA-3-COOH	21

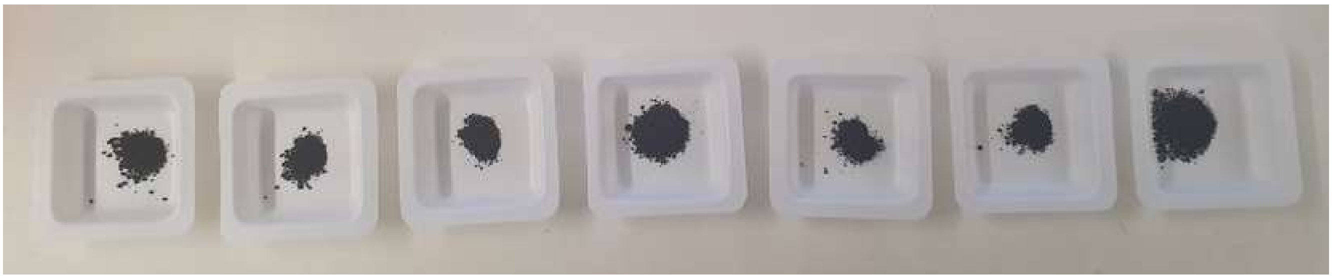

Figure S1: Powders left to right; PTPA, PTPA-3, PTPA-9, PTPA-15 PTPA-50, PAni and PTPA-3-COOH

Table S1: Feed ratios for formation of the extended networks

	tris(4-bromophenyl)amine	p-phenylenediamine	1,4 -dibromobenzene	Pd(dba)2 (dba=dibenzylideneacetone	2-dicyclohexylphosphino-2',4',6'-triisopropylbiphenyl	sodium tert-butoxide	2,5-dibromobenzoic acid
PTPA	362 mg	54 mg	-	29 mg	52 mg	384 mg	-
PTPA-3	120 mg	324 mg	531 mg	530 mg	127 mg	384 mg	-
PTPA-9	60 mg	406 mg	797 mg	266 mg	310 mg	384 mg	-
PTPA-15	60 mg	649 mg	1327 mg	609 mg	224 mg	384 mg	-
PTPA-50	10 mg	331 mg	708 mg	138 mg	262 mg	384 mg	-
PAni	-	54 mg	118 mg	29 mg	52 mg	384 mg	-
PTPA-3-COOH	121 mg	324 mg	-	531 mg	127 mg	577 mg	630 mg

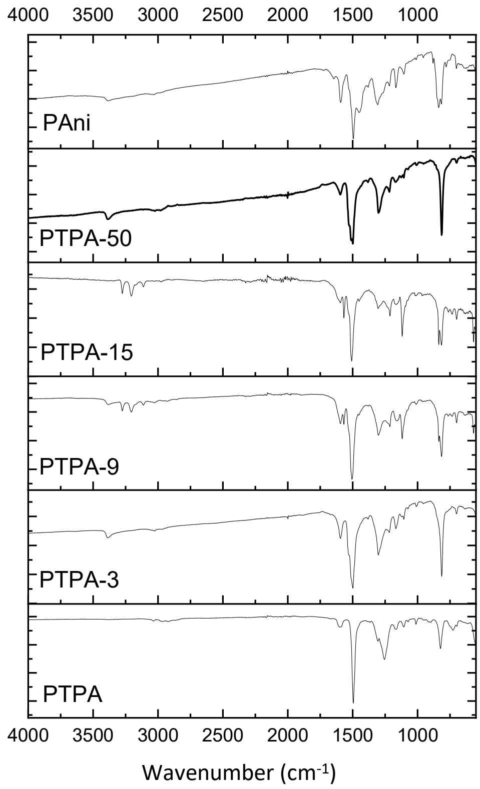

Figure S2: FTIR spectra of extended networks

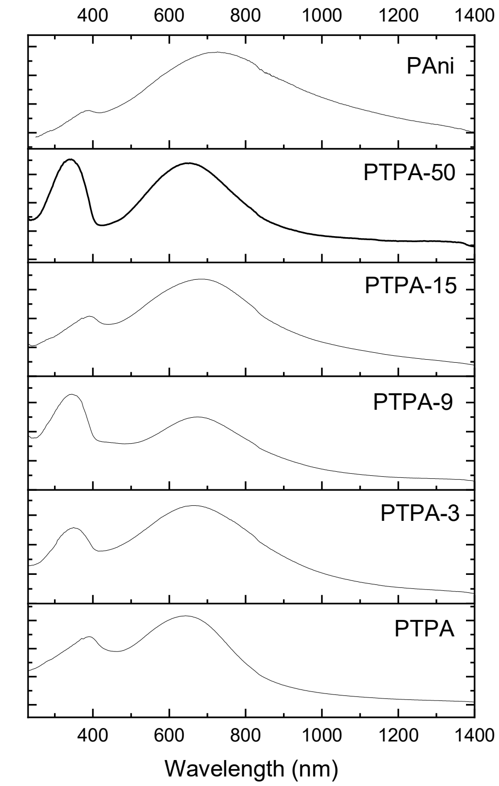

Figure S3: Solid state UV-vis spectra of extended networks

Table S2: Decomposition temperatures (5% and 10% loss in weight) as determined by TGA for extended networks

Sample	5% loss in weight (°C)	10% loss in weight (°C)
PTPA	270	390
PTPA-3	280	347
PTPA-9	227	310
PTPA-15	231	270
PTPA-50	232	323
PAni	193	260
PTPA-3-COOH	247	416

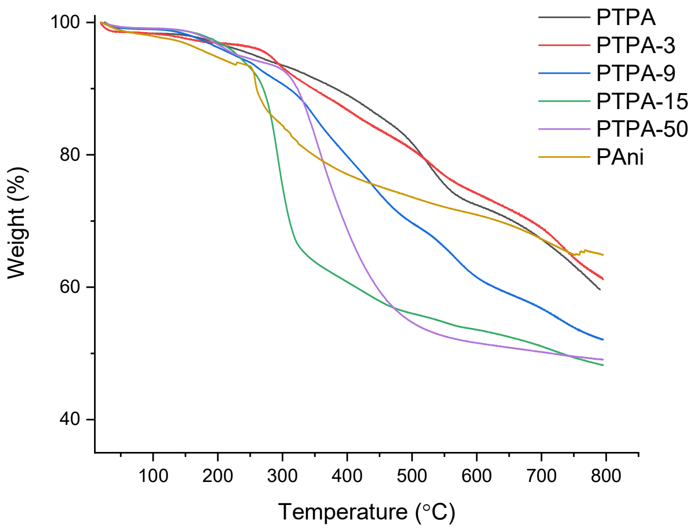

Figure S4: TGA of the extended networks

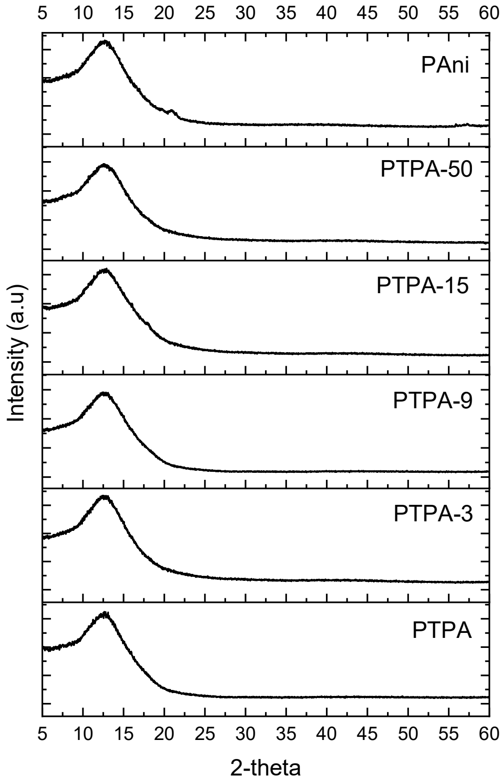

Figure S5: XRD analysis of the extended networks

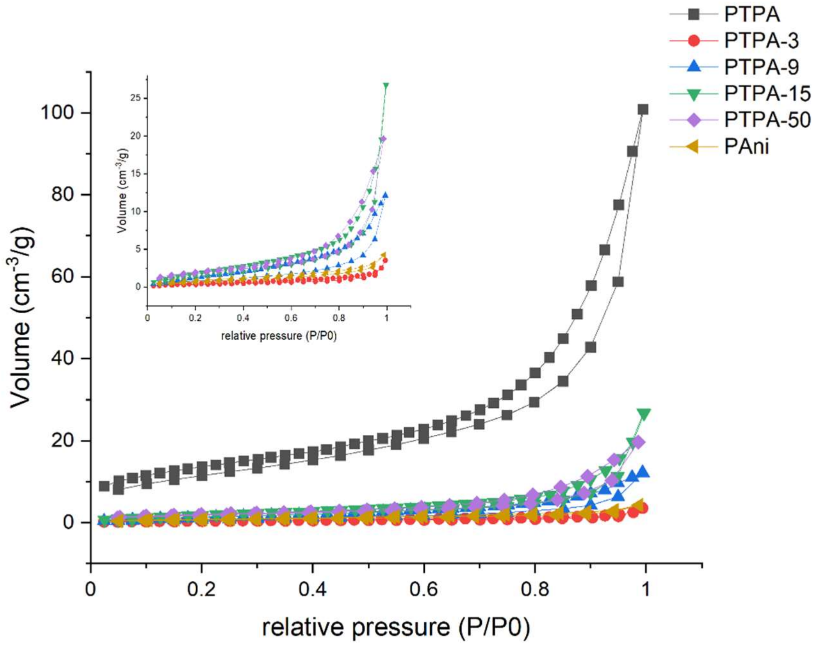

Figure 6a: Nitrogen BET isotherms of the extended networks collected at 273 K

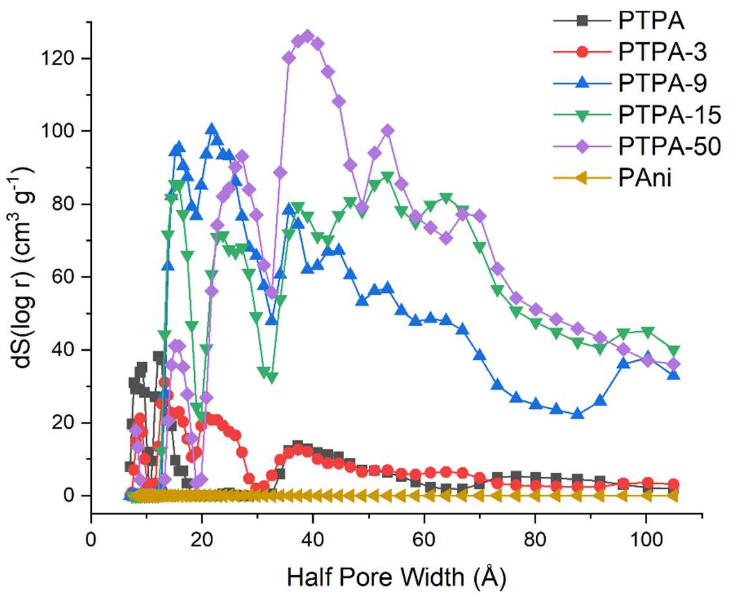

Figure 6b: DFT pore size distribution of the extended networks

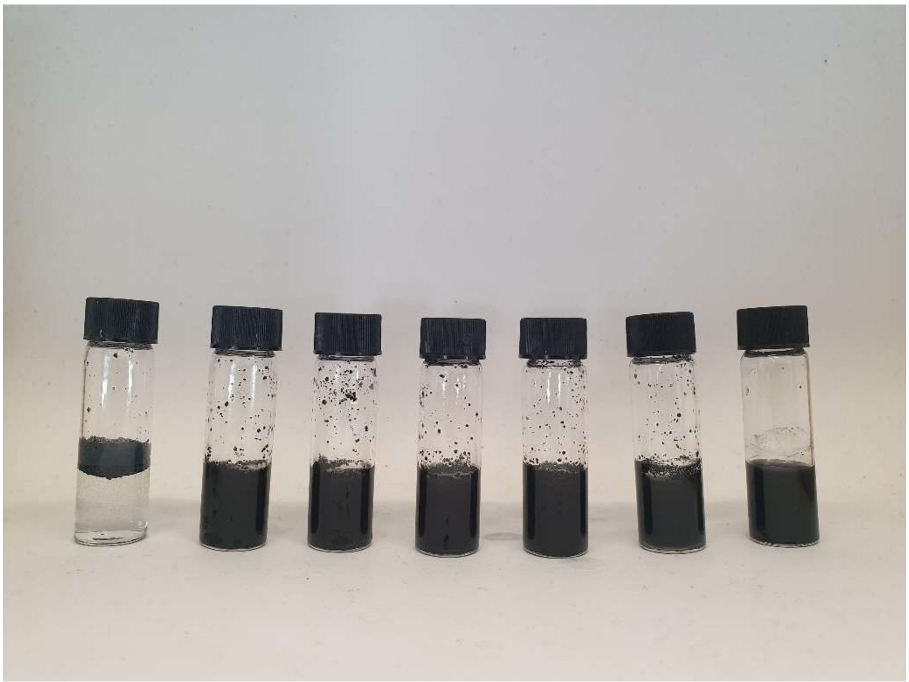

Figure S7: Aqueous dispersions of PTPA, PTPA-3, PTPA-9, PTPA-15, PTPA-50, PAni, PTPA-3-COOH (left to right)

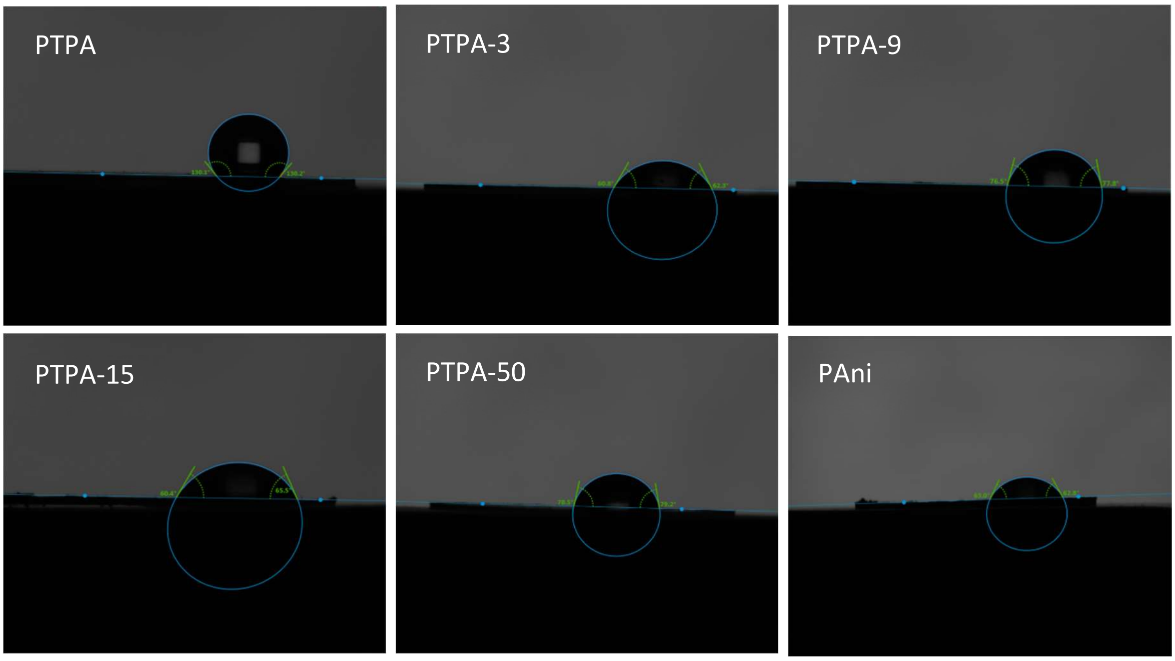

Figure S8a: Contact angle images of the extended networks

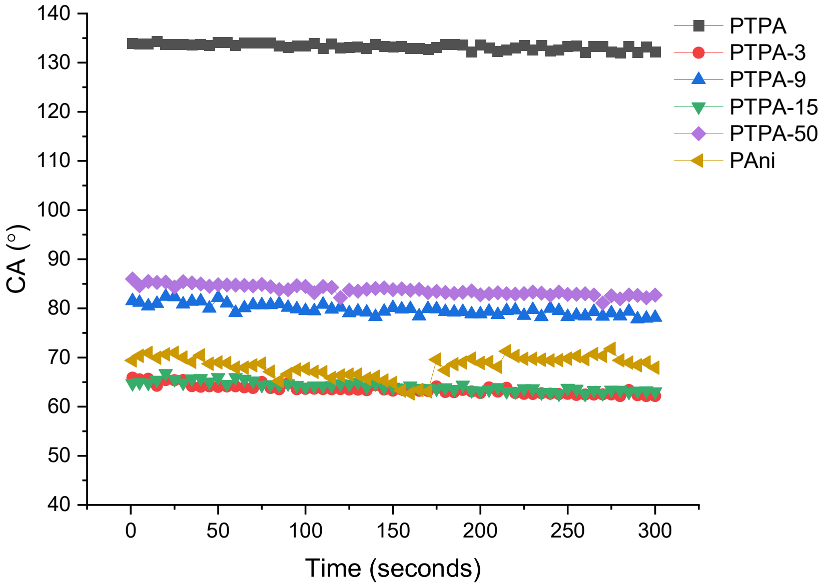

Figure S8b: Contact angle measurements of the extended networks over 300 seconds

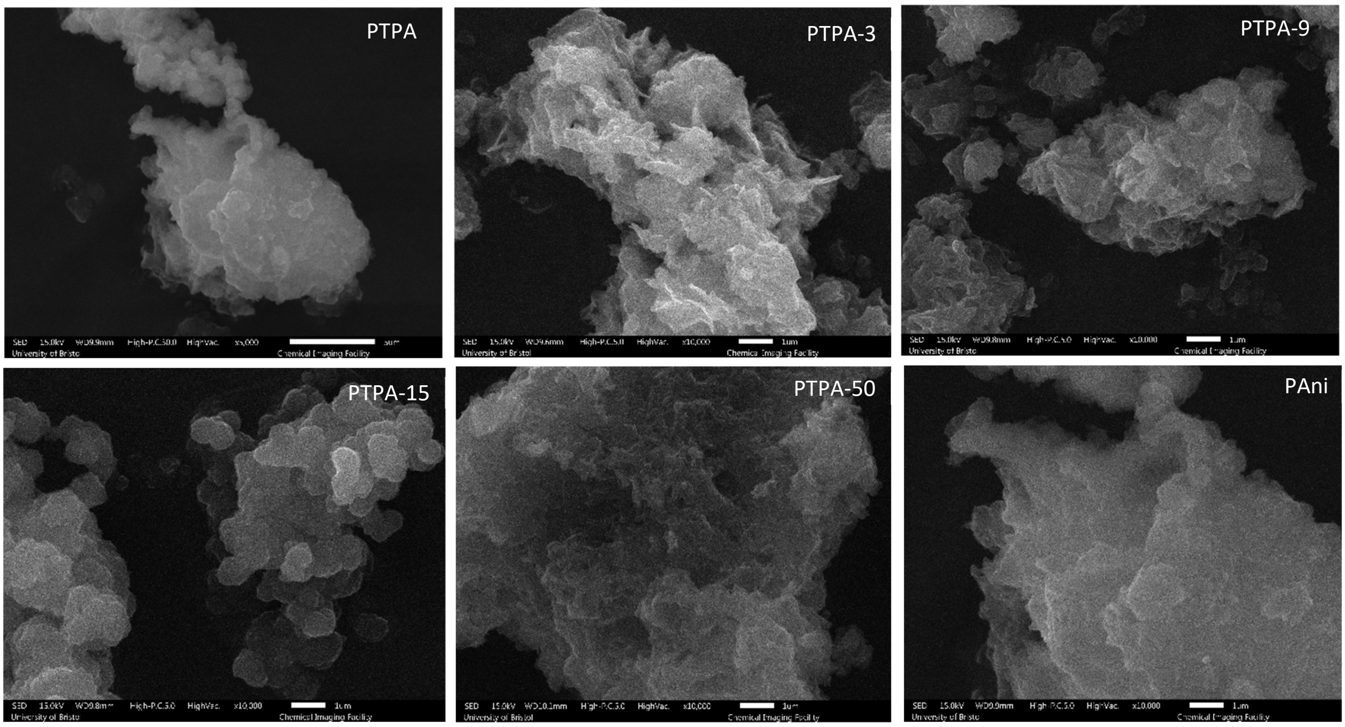

Figure S9: SEM images of the extended networks

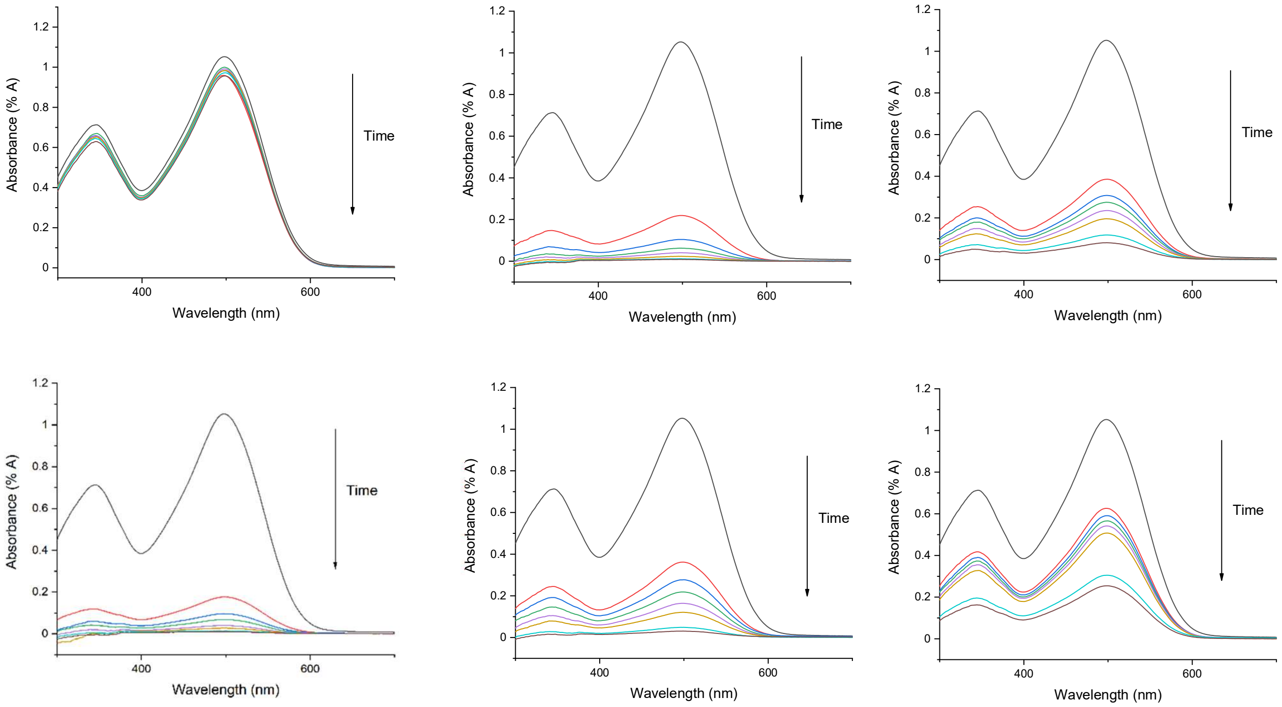

Figure S10a: UV-vis spectra showing the adsorption of congo red dye by PTPA, PTPA-3, PTPA-9 (top left to right), PTPA-15, PTPA-50, PAni (bottom left to right)

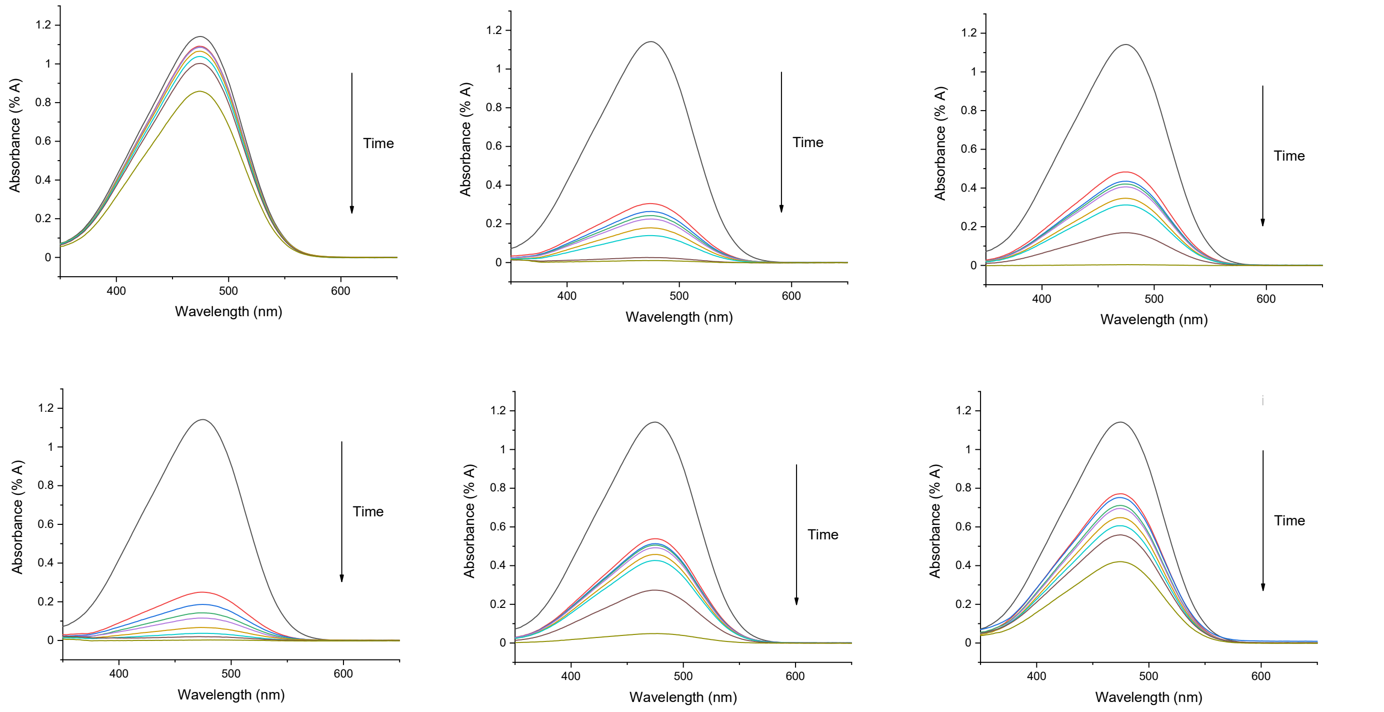

Figure S10b: UV-vis spectra showing the adsorption of ethyl orange dye by PTPA, PTPA-3, PTPA-9 (top left to right), PTPA-15, PTPA-50, PAni (bottom left to right)

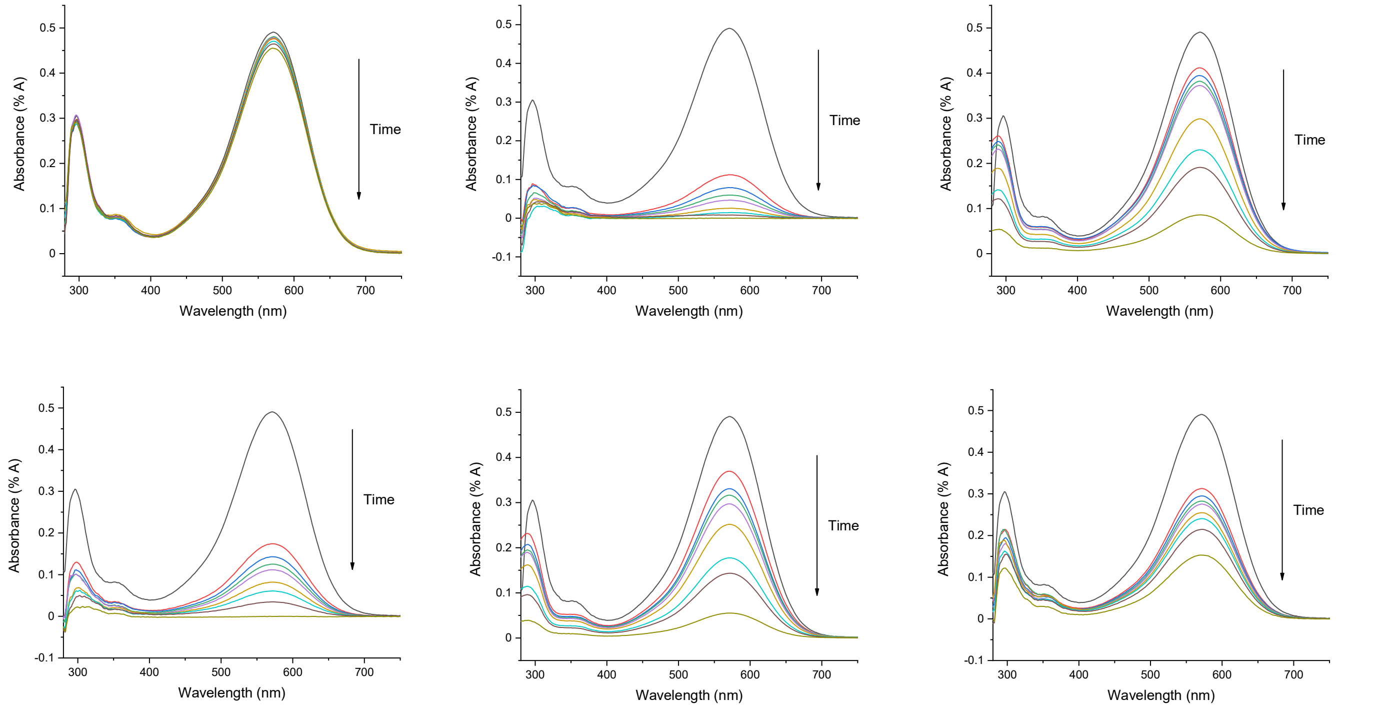

Figure S10c: UV-vis spectra showing the adsorption of Acid blue dye by PTPA, PTPA-3, PTPA-9 (top left to right), PTPA-15, PTPA-50, PAni (bottom left to right)

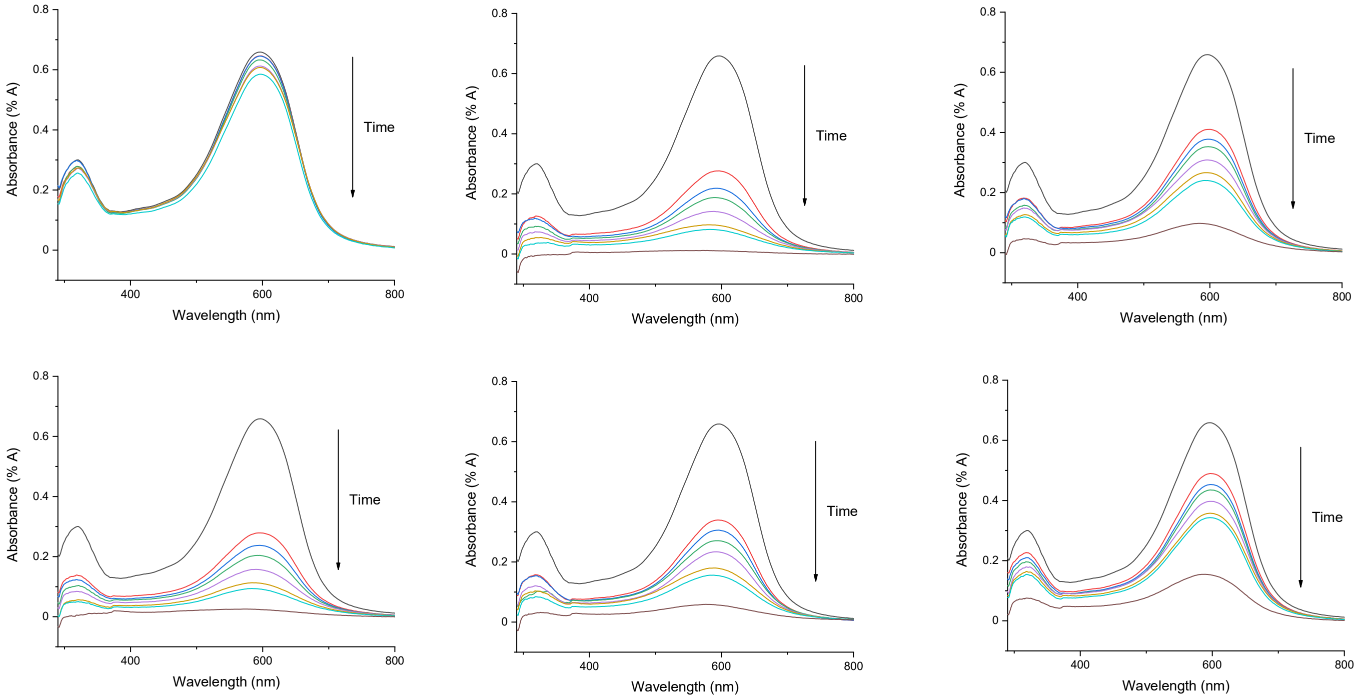

Figure S10d: UV-vis spectra showing the adsorption of direct blue dye by PTPA, PTPA-3, PTPA-9 (top left to right), PTPA-15, PTPA-50, PAni (bottom left to right)

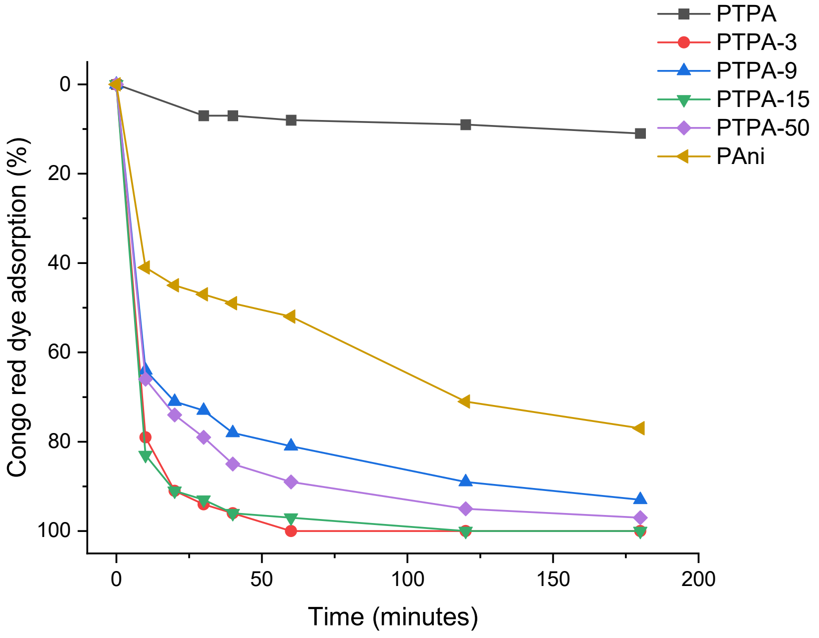

Figure S11a: Adsorption of congo red dye over time by extended networks

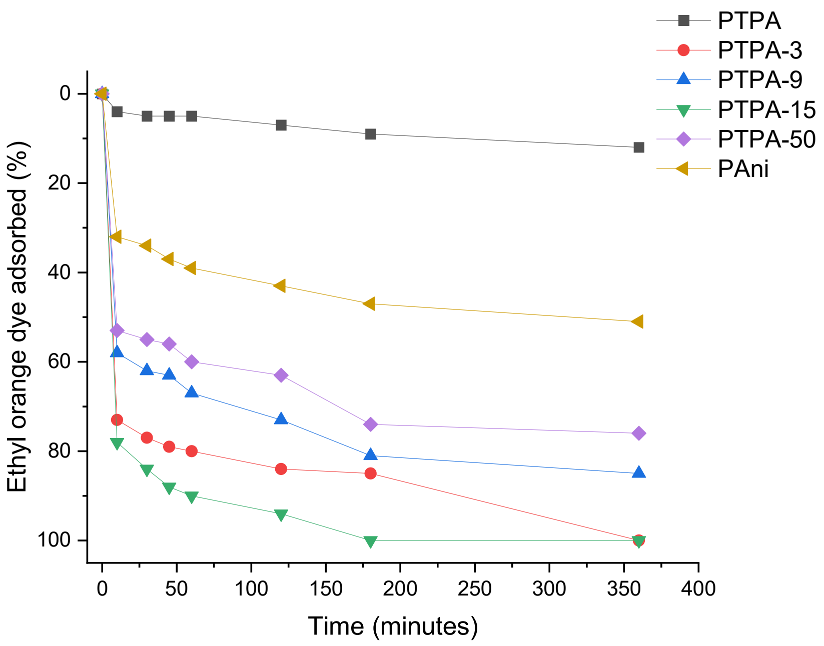

Figure S11b: Adsorption of ethyl orange dye over time by extended networks

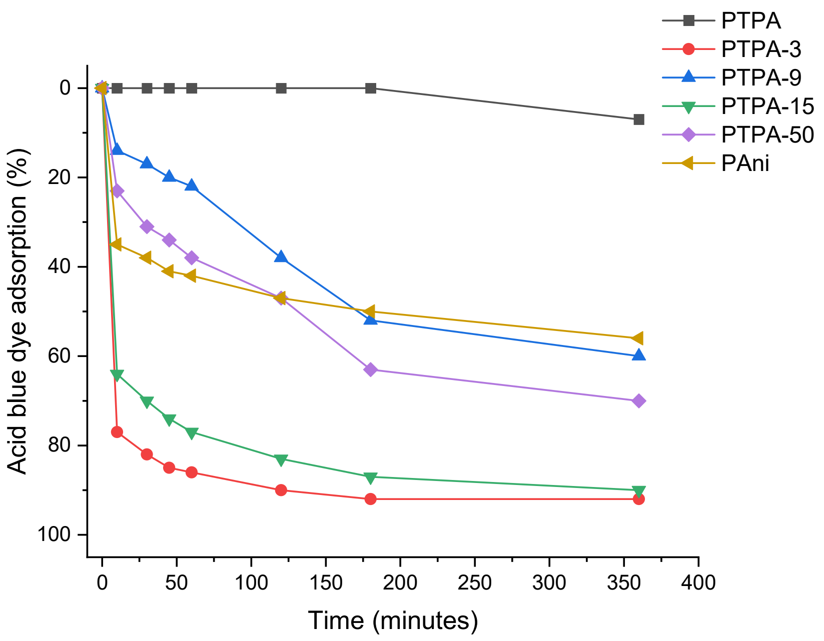

Figure S11c: Adsorption of acid blue dye over time by extended networks

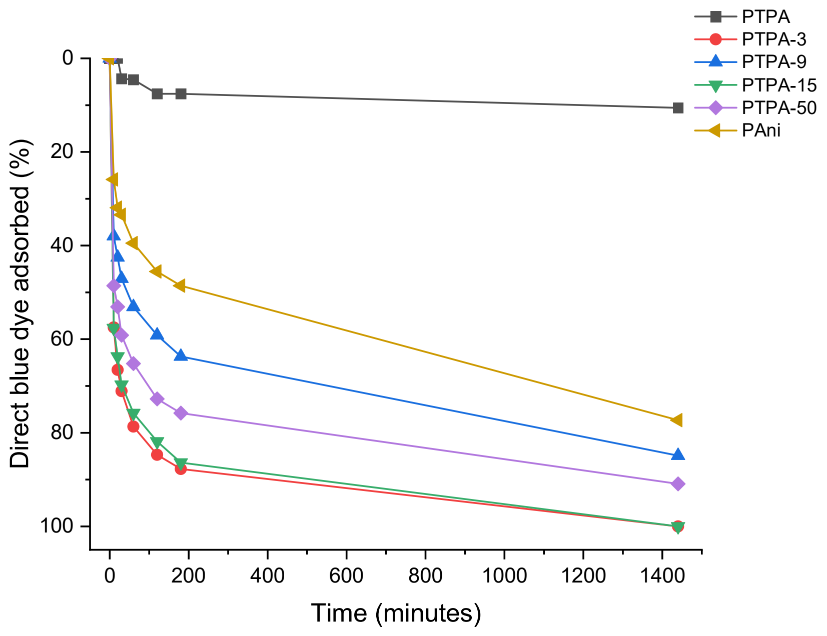

Figure S11d: Adsorption of direct blue dye over time by extended networks

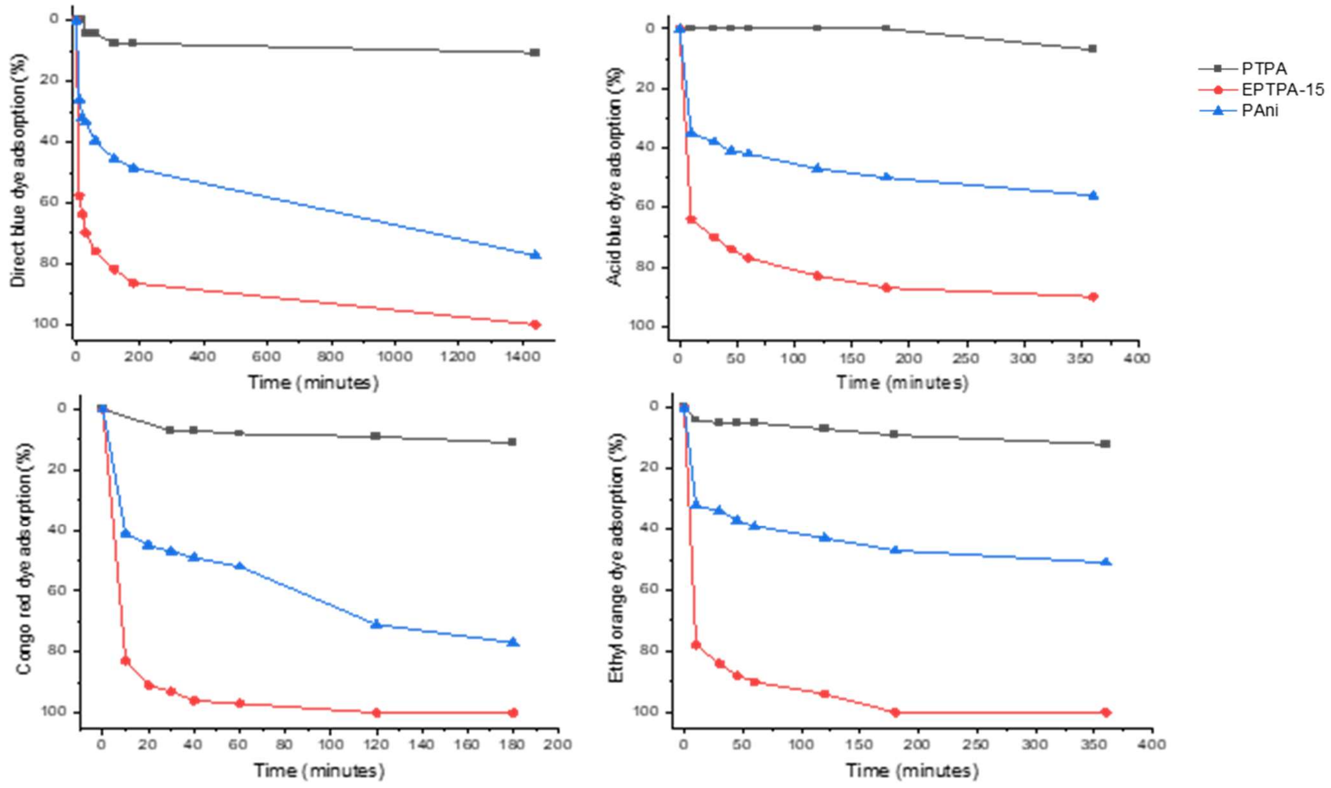

Figure S12: Direct comparison of the dye adsorption permformance of PTPA and Pani against the optimised extended network PTPA-15

Table S3: Coefficient of correlation (R^2) values for pseudo zero, first and second order absorption rates, values were calculated via fitting [A] against [A]calc in each rate equation, [A]calc was derived from the three rate laws under the graph, highlighted in green are the most significant fits for each dye absorbed by a particular network.

	Direct Blue				Absolute Blue		
	Zero Order	First Order	Second Order		Zero Order	First Order	Second Order
**PTPA**	0.532007	0.206254	0.233363	**PTPA**	0.51734	0.349638	0.527934
**3 EXT**	0.18648	0.183648	0.612475	**3 EXT**	0.301386	0.684788	0.825916
**9 EXT**	0.448143	0.964828	0.845245	**9 EXT**	0.200177	0.879975	0.343989
**15 EXT**	0.443521	0.994551	0.894275	**15 EXT**	0.286119	0.684702	0.241763
**50 EXT**	0.144969	0.929576	0.722404	**50 EXT**	0.134567	0.803851	0.289274
**Pani**	0.337337	0.990425	0.919121	**Pani**	0.10287	0.660421	0.946575

	Ethyl Orange				Congo Red		
	Zero Order	First Order	Second Order		Zero Order	First Order	Second Order
**PTPA**	0.921179	0.349638	0.943723	**PTPA**	0.439727	0.44721	0.454991
**3 EXT**	0.157915	0.22305	0.950787	**3 EXT**	0.511726	0.898537	0.990203
**9 EXT**	0.342018	0.879975	0.469313	**9 EXT**	0.184988	0.848207	0.423968
**15 EXT**	0.335205	0.732935	0.405242	**15 EXT**	0.286119	0.684702	0.241763
**50 EXT**	0.160269	0.834944	0.47561	**50 EXT**	0.134567	0.803851	0.289274
**Pani**	0.396582	0.963122	0.942197	**Pani**	0.10287	0.660421	0.946575

Zero order; $[A]=[A]_0-kt$

First order; $\ln[A]=\ln[A]_0-kt$

Second order; $1/[A]=1/[A]_0+kt$

Whereby t = time, k = rate constant [A] = absorption, $[A]_0$ = absorption at time 0. Values of k were obtained by plotting $[A]_0$ and $[A]_{eq}$ (whereby $[A]_{eq}$ is the absorbance value at equilibrium taken from experimental data) and fitting straight line graphs for the three equations given above.

Table S4: Absorption capacities of Activated carbon of the dyes of interest

Toxic dye	Activated carbon adsorption capacity (mg/g)
**CR**	100[1]
**AB**	89[2]
**DB**	135[3]
**EO**	35[4]

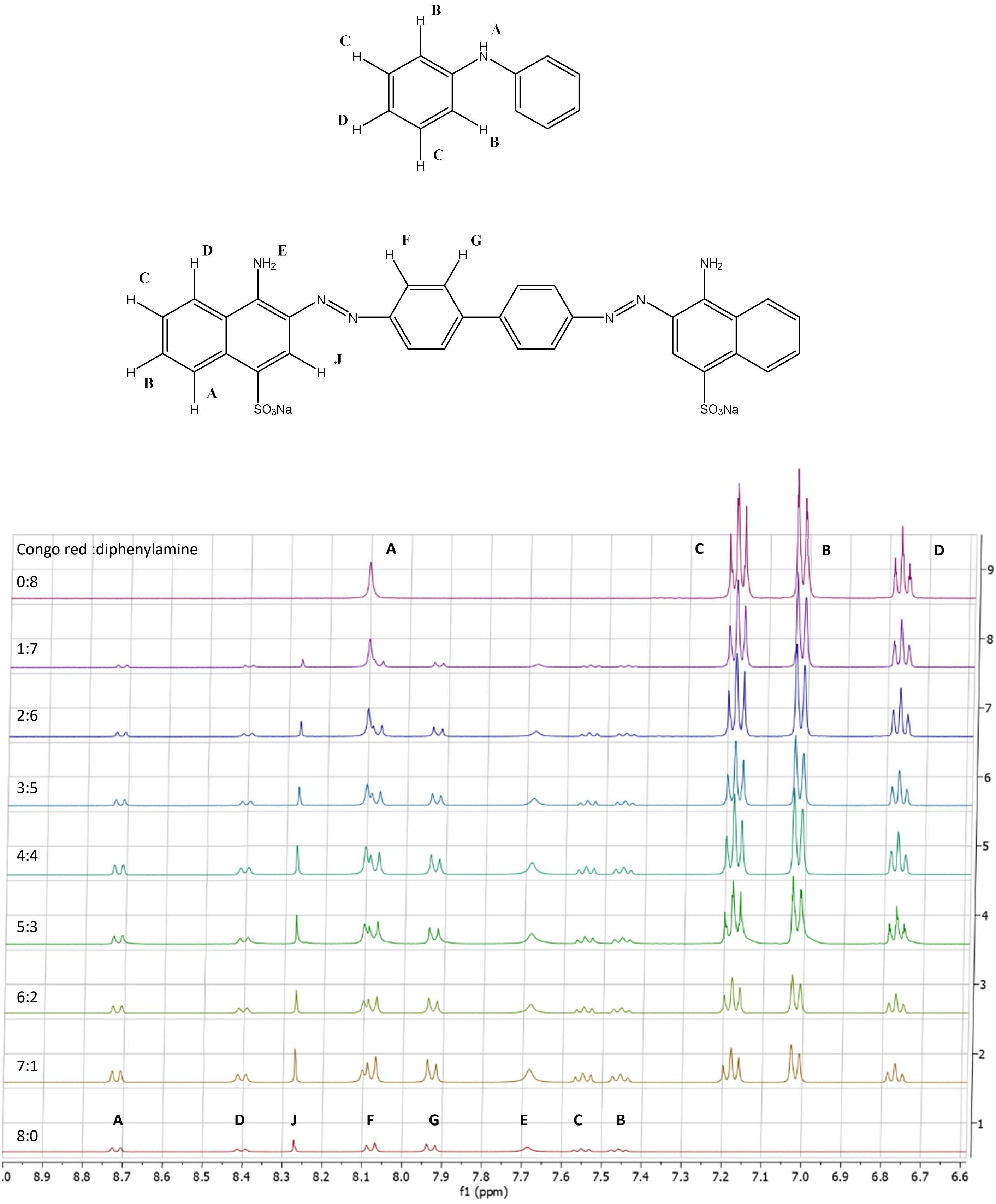

Figure S13a: 1H NMR binding study of the interactions between congo red (bottom structure) and diphenylamine (top structure) in DMSO

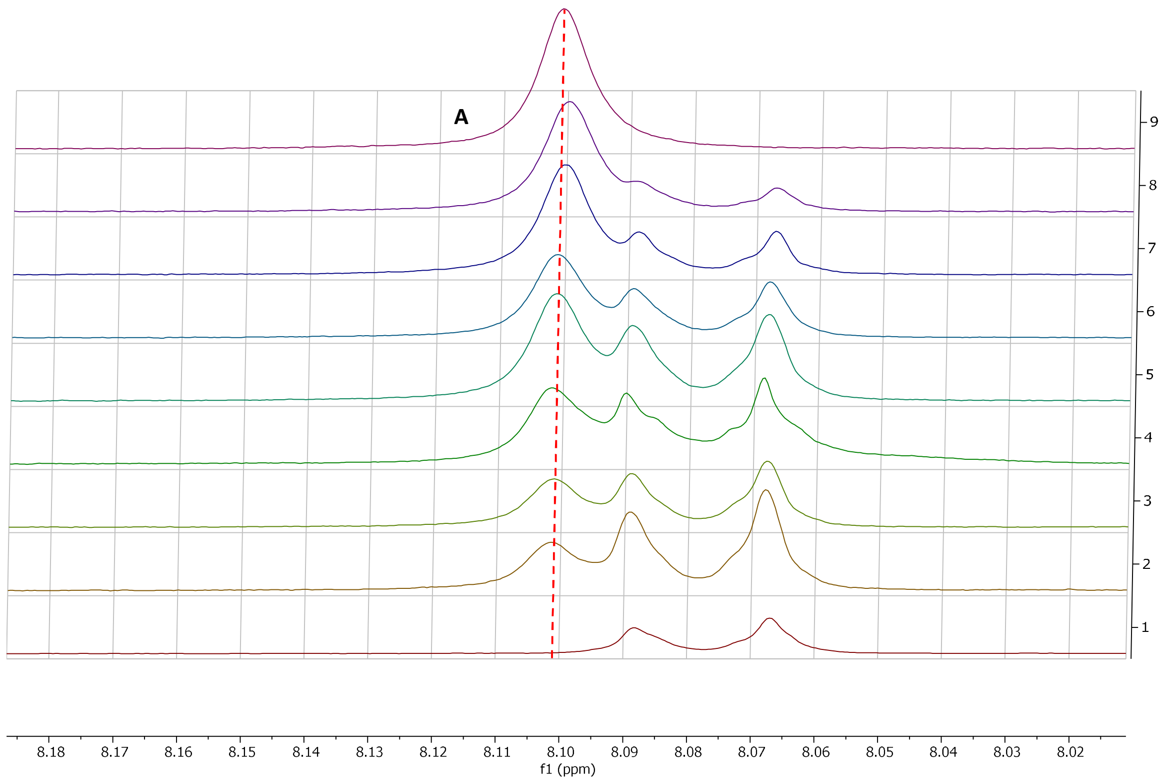

Figure S13b: ^{1}H NMR peak assigned to congo red amine hydrogen shifting with ratio to diphenylamine (Figure S13a)

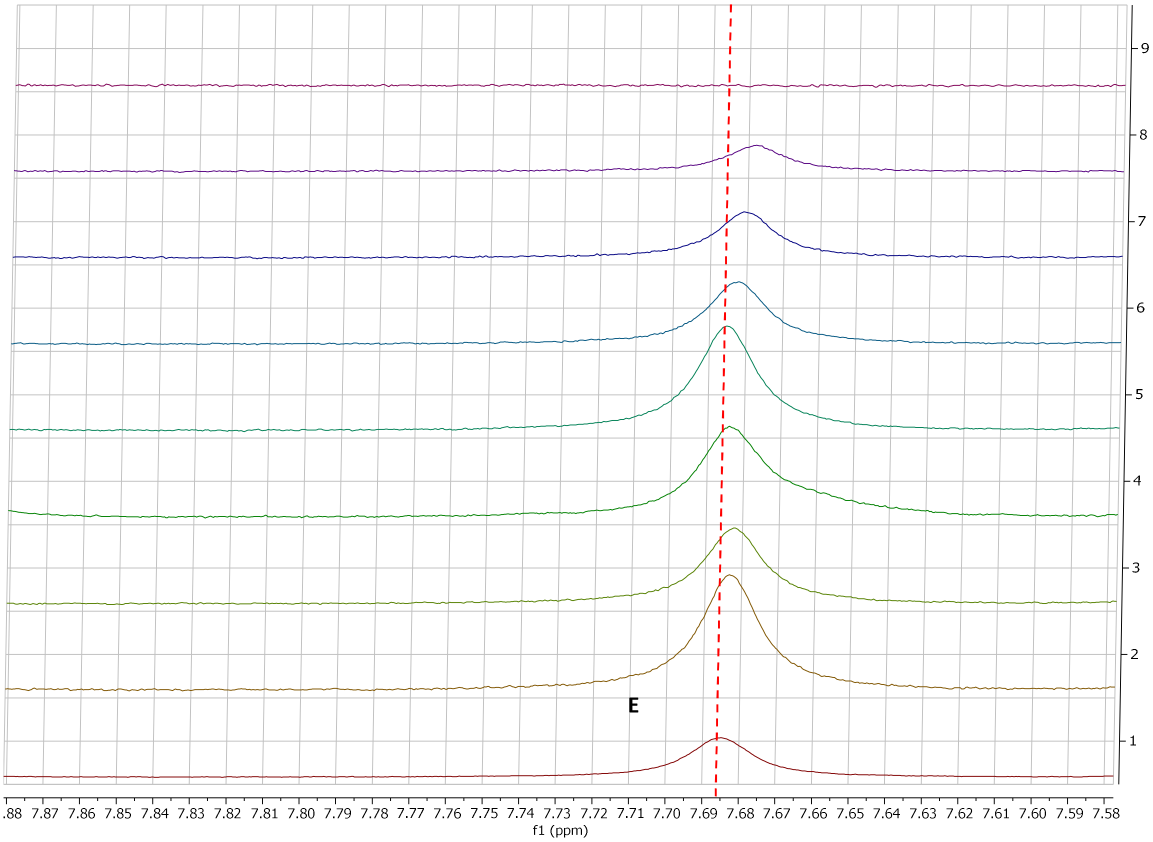

Figure S13c: ^{1}H NMR peak assigned to diphenylamine amine hydrogen shifting with ratio to diphenylamine (Figure S13a)

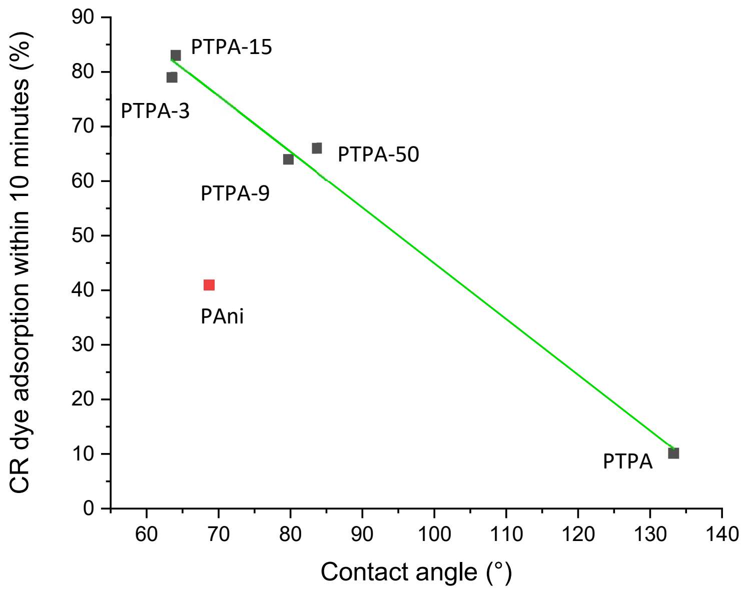

Figure S14; CR dye absorption as a function of contact angle

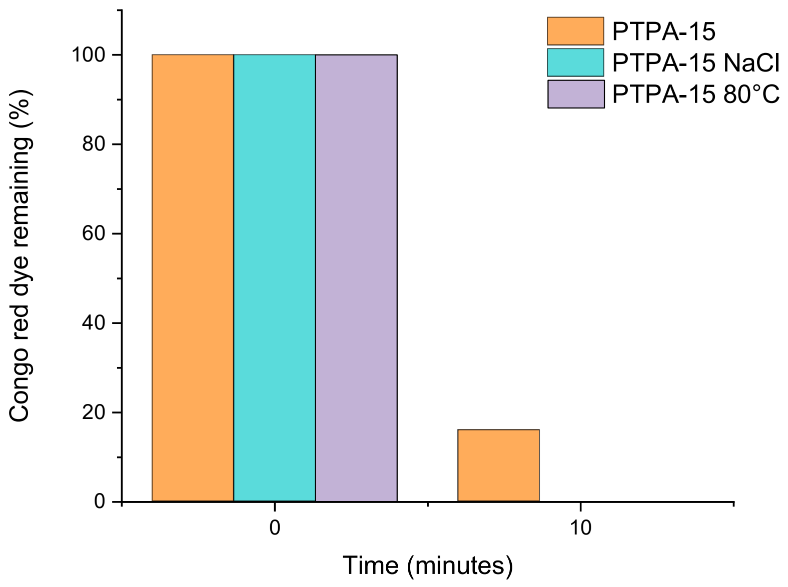

Figure S15. CR adsorption by **PTPA-15** over varying aqueous conditions

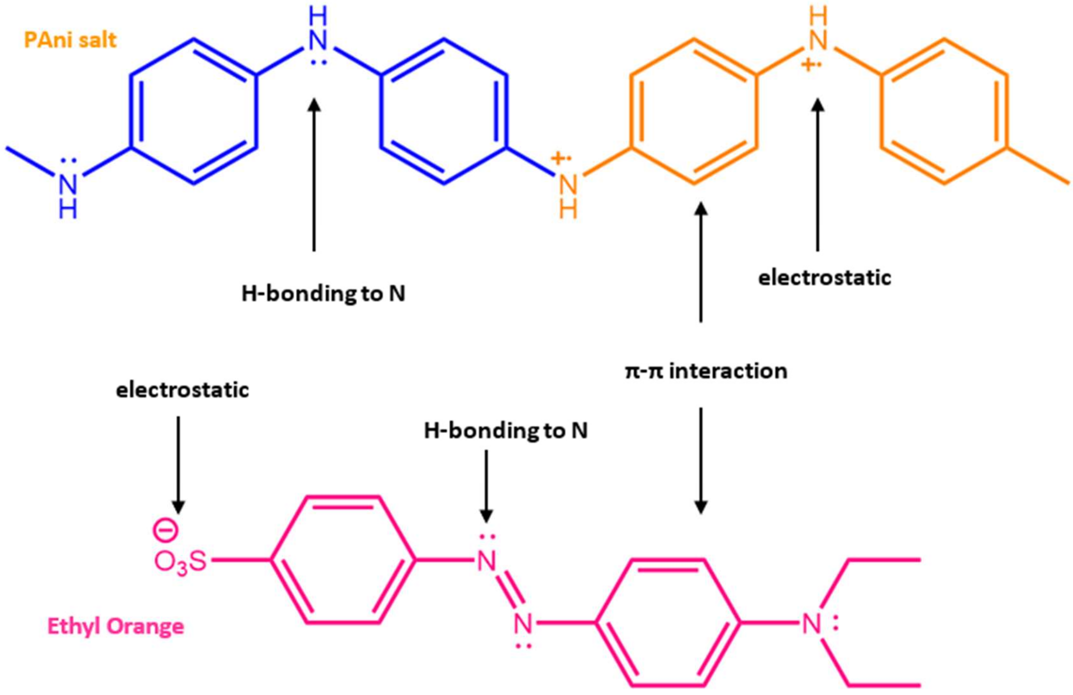

Figure S16: Theorised interactions between the EO dye and Polyanaline like networks.

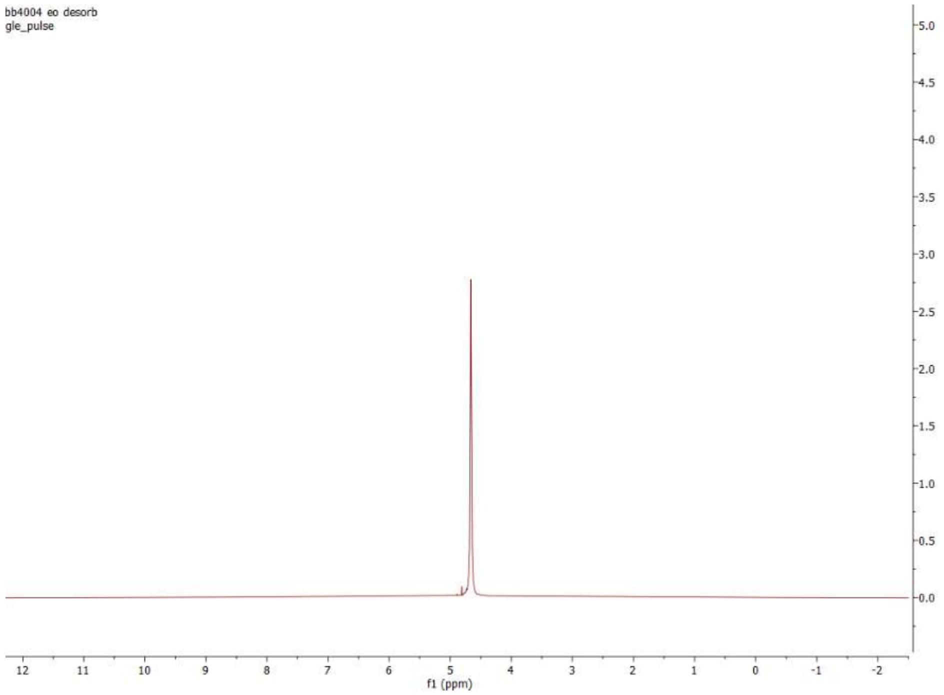

Figure S17: ^{1}H NMR analysis of D_2O elute after 5th cycle from gel:**PTPA-15** EO absorption tests.

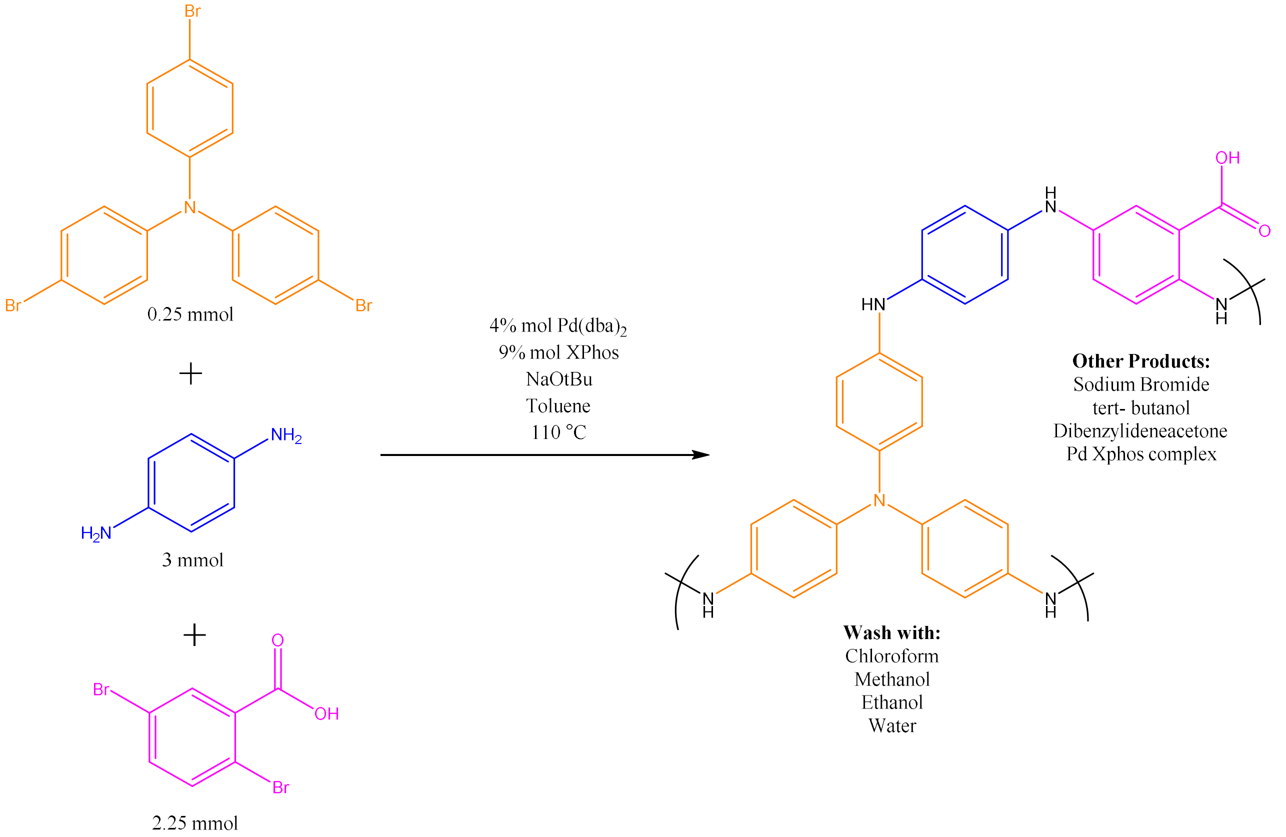

Scheme S1: Synthetic route to PTPA-3-COOH

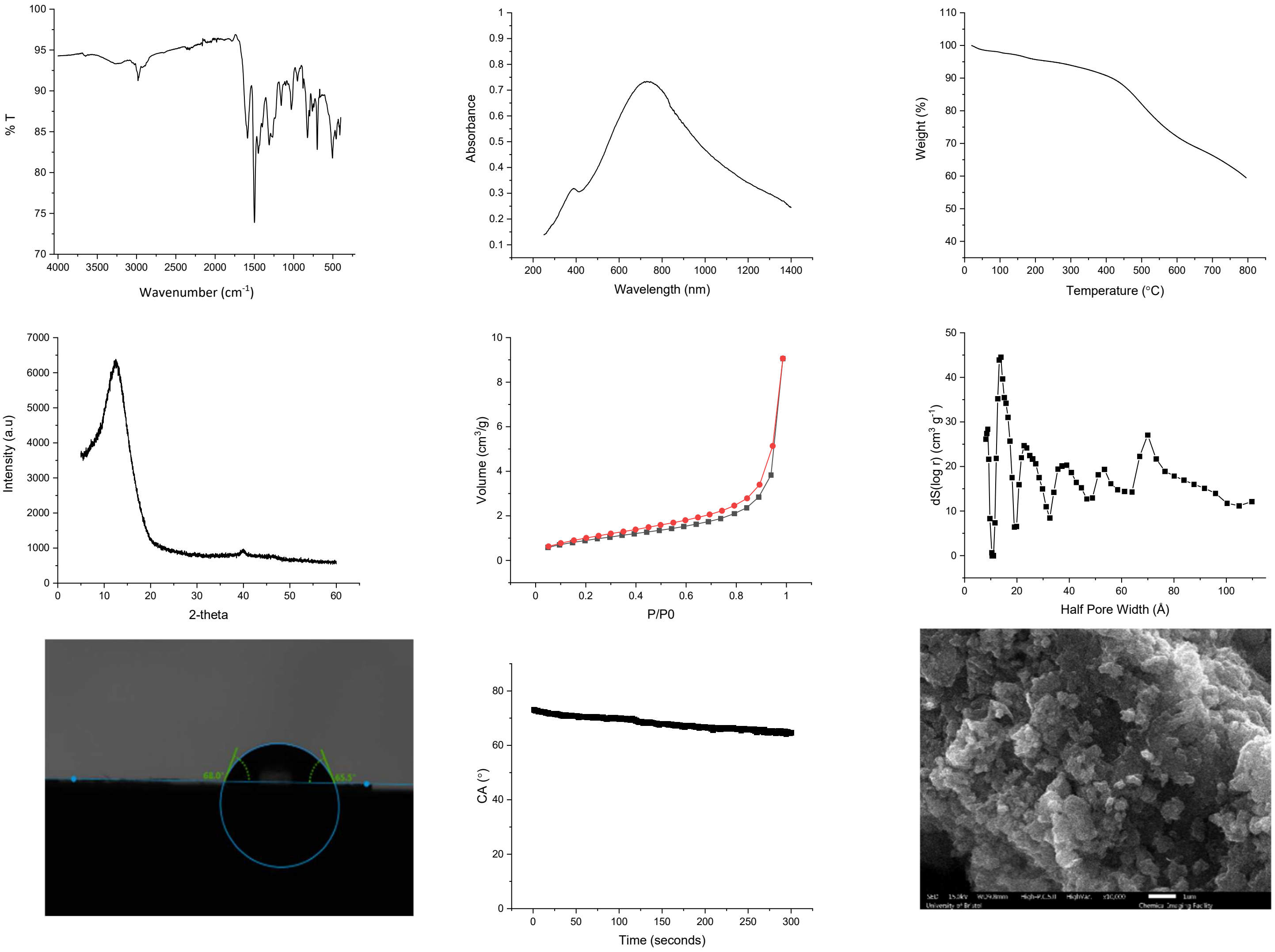

Figure S18: Characterisation of PTPA-3-COOH. Top row left to right: FTIR, UV-vis, TGA. Middle row left to right: XRD, nitrogen BET isotherm, DFT pore size distribution. Bottom row left to right: contact angle image, contact angle over 300 seconds, SEM image.

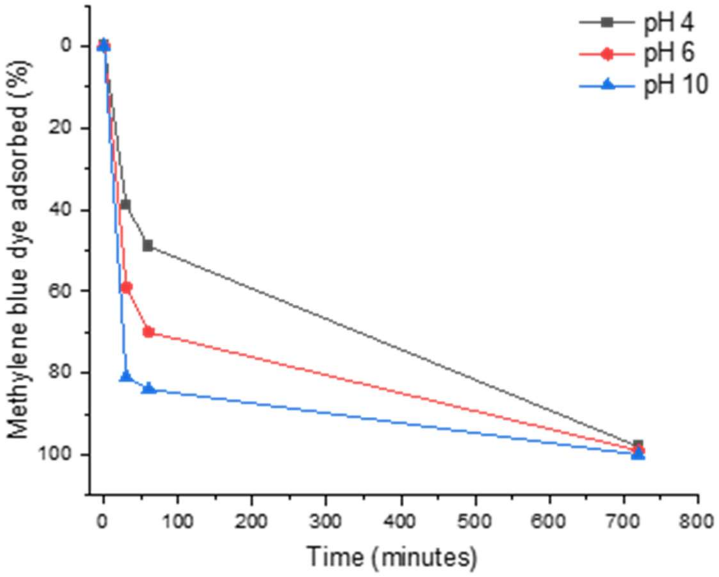

Figure S19: Adsorption of methylene blue dye over time by PTPA-3-COOH at pH 4, pH 6, and pH 10

References

1, R. Lafi, I. Montasser and A. Hafiane, Adsorption Science & Technology, 2019, 37, 160–181.

2, F. Ali, N. Ali, I. Bibi, A. Said, S. Nawaz, Z. Ali, S. M. Salman, H. M. N. Iqbal and M. Bilal, Case Studies in Chemical and Environmental Engineering, 2020, 2, 100040.

3, L. D. T. Prola, F. M. Machado, C. P. Bergmann, F. E. de Souza, C. R. Gally, E. C. Lima, M. A. Adebayo, S. L. P. Dias and T. Calvete, Journal of Environmental Management, 2013, 130, 166–175.

4, S. Dutta, B. Gupta, S. K. Srivastava and A. K. Gupta, Mater. Adv., 2021, 2, 4497–4531.
